# Mid-Term and Long-Term Clinical Events with Conduction System Pacing Versus Biventricular Pacing in HFrEF with Wide QRS: A Systematic Review and Meta-Analysis of Randomized Controlled Trials with Trial Sequential Analysis

**DOI:** 10.3390/jcm15208087

**Published:** 2026-10-18

**Authors:** Juan Munoz-Moreno, Carlos Diaz-Arocutipa, Carlos Espiche-Salazar, Jacob McAuliffe, Raymond L. Benza, Amin Yehya, Johanna Contreras, Erich L. Kiehl

**Affiliations:** 1 Department of Internal Medicine, Macon and Joan Brock Virginia Health Sciences at Old Dominion University, Norfolk, VA 23507, USA; 2 Unidad de Revisiones Sistemáticas y Meta-Análisis (URSIGET), Vicerrectorado de Investigación, Universidad San Ignacio de Loyola, Lima 15024, Peru; cdiazar@usil.edu.pe; 3 Department of Cardiology, Bassett Medical Center, Cooperstown, NY 13326, USA; carlos.espichesalazar@bassett.org; 4 Department of Cardiology, Inova Schar Heart and Vascular Institute, Falls Church, VA 22042, USA; jacob.mcauliffe@inova.org; 5 Department of Cardiology, Macon and Joan Brock Virginia Health Sciences at Old Dominion University, Norfolk, VA 23507, USA; rbenza@odu.edu (R.L.B.); axyehya@sentara.com (A.Y.); elkiehl@sentara.com (E.L.K.); 6 Advanced Heart Failure Center, Sentara Heart Hospital, Norfolk, VA 23507, USA; 7 Department of Medicine, Division of Cardiology, Icahn School of Medicine, Mount Sinai Fuster Heart Hospital, New York, NY 10029, USA; johanna.contreras@mountsinai.org; 8 Department of Electrophysiology, Sentara Medical Group, Sentara Health, Virginia Beach, VA 23454, USA

**Keywords:** biventricular pacing, cardiac resynchronization therapy, conduction system pacing, heart failure with reduced ejection fraction, meta-analysis, randomized controlled trials

## Abstract

**Background/Objectives:** Conduction system pacing (CSP) has emerged as a more physiological alternative to biventricular pacing (BiVP) in heart failure with reduced ejection fraction (HFrEF) and wide QRS, but recent randomized controlled trials (RCTs) have yielded discordant results. This meta-analysis aimed to compare CSP with BiVP for clinical events and functional recovery in HFrEF. **Methods:** MEDLINE, Embase, and Cochrane Library were searched for RCTs through 15 April 2026. Primary outcomes were all-cause mortality (ACM) and heart failure hospitalization (HFH) at mid-term (6–12 months) and long-term (>12 months) follow-up. Random-effects meta-analyses and Trial Sequential Analysis (TSA) were performed. **Results:** Eleven RCTs comprising 12 reports and 1073 patients were included. CSP showed no significant difference in mid-term ACM (RR 0.72; 95% CI 0.21 to 2.49; *p* = 0.605) or HFH (RR 0.97; 95% CI 0.52 to 1.81; *p* = 0.918). At long-term follow-up (three RCTs; n = 312), CSP showed a non-significant trend toward ACM reduction (RR 0.34; 95% CI 0.11 to 1.06; *p* = 0.063; low-certainty evidence) and a significant, TSA-supported reduction in HFH (RR 0.30; 95% CI 0.16 to 0.58; *p* < 0.001; moderate-certainty evidence). At 6 months, CSP improved LVEF (MD 2.84%; 95% CI 0.74 to 4.93; *p* = 0.008) and reduced QRS duration (MD −9.97 ms; 95% CI −18.57 to −1.37; *p* = 0.023), with NYHA improvement at 12 months (MD −0.31; 95% CI −0.50 to −0.11; *p* = 0.002). **Conclusions:** In this meta-analysis of RCTs predominantly evaluating left bundle branch area pacing, CSP was associated with a lower risk of long-term HFH compared with BiVP in HFrEF with a wide QRS, although the evidence remains limited by the small number of available trials. The observed trend toward lower long-term ACM should be considered hypothesis-generating. Larger, adequately powered RCTs with longer follow-up are needed to confirm these findings and determine whether CSP provides sustained clinical benefit over BiVP.

## 1. Introduction

Biventricular pacing (BiVP) is the established standard for cardiac resynchronization therapy (CRT) in heart failure with reduced ejection fraction (HFrEF) with wide QRS, significantly reducing all-cause mortality (ACM), heart failure hospitalization (HFH), and promoting left ventricular reverse remodeling across decades of randomized evidence, as it is reflected in multi-societal guidelines [1,2]. However, approximately one-third of patients fail to achieve meaningful clinical or echocardiographic response, successful left ventricular lead delivery is not always feasible due to unfavorable coronary sinus anatomy, elevated branch thresholds, or phrenic nerve stimulation, and non-physiological epicardial activation generates residual ventricular dyssynchrony that limits CRT response even when implantation is successful [3,4,5].

Conduction system pacing (CSP) has emerged as a more physiological CRT modality by engaging the native His–Purkinje network to restore synchronous ventricular activation, in contrast to the epicardial stimulation inherent to BiVP [6,7,8]. His-bundle pacing (HBP), the pioneering CSP strategy, established a mechanistic proof of concept but was limited by high, unstable pacing thresholds and a steep learning curve [9,10]. Left bundle branch area pacing (LBBAP), targeting the left bundle branch and its proximal arborizations via deep septal lead deployment, has emerged as the dominant CSP modality with lower and more stable capture thresholds, higher procedural success rates, and greater technical reproducibility [9,11]. Reflecting this evidence, the 2023 HRS/APHRS/LAHRS guideline assigned a Class IIa recommendation to CSP when effective CRT cannot be achieved with BiVP due to anatomical or functional criteria [1], while the 2025 ESC clinical consensus advises CSP as rescue therapy when coronary sinus lead implantation is unsuccessful and considers it may be appropriate as an alternative to BiVP in selected candidates [2].

A previous meta-analysis reported significant improvements in QRS duration, left ventricular ejection fraction (LVEF), and New York Heart Association (NYHA) functional class, favoring CSP over BiVP, with a predominantly six-month follow-up [12]. However, the limited sample size and short observational horizon may have been insufficient to detect differences in clinical outcomes. More recently, several large randomized controlled trials (RCTs) have been published that may strengthen the power of pooled estimates [13,14,15,16,17], while some have yielded discordant findings [15,16], collectively justifying an updated cumulative evidence synthesis. Therefore, we aimed to perform an updated systematic review and meta-analysis restricted to RCT-level evidence, pooling more than 2.5 times the prior sample size, with several prespecified enhancements: analysis of clinical events stratified by mid-term versus long-term follow-up; functional and procedural outcomes analyzed separately at 6 and 12 months, maintaining subgroup analysis by CSP modality (LBBAP versus HBP); and Trial Sequential Analysis (TSA) for outcomes. Additionally, unlike the prior analysis, we excluded one RCT that enrolled patients with narrow QRS [18], and outcome data derived from mixed-model repeated-measures in another [19], consistent with established standards for the validity of pooled effect estimates.

## 2. Methods

### 2.1. Protocol, Registration, and Study Eligibility Criteria

This study protocol was registered in the International Prospective Register of Systematic Reviews (PROSPERO) database (CRD420261371613). The systematic review and meta-analysis were guided by the Preferred Reporting Items for Systematic Reviews and Meta-Analyses 2020 statement [20].

Eligible studies were RCTs comparing CSP—encompassing LBBAP and HBP—versus BiVP in adults (≥18 years) with HFrEF and wide QRS duration, with no restriction applied regarding QRS morphology (left bundle branch block (LBBB) or non-specific intraventricular conduction delay) or underlying etiology of heart failure (ischemic or nonischemic cardiomyopathy). Observational studies, case series, conference abstracts without peer-reviewed full-text publications, and studies with overlapping patient populations or lacking a comparator group were excluded.

### 2.2. Search Strategy and Study Identification

Systematic searches were conducted in MEDLINE (via PubMed), Embase, and the Cochrane Central Register of Controlled Trials from inception through April 15, 2026, using terms related to heart failure, cardiac resynchronization therapy, biventricular pacing, and conduction system pacing (full search strategies available in Supplementary Material, Table S1). No language or date restrictions were applied. Reference lists of included studies and relevant systematic reviews were hand-searched for additional eligible records.

### 2.3. Study Selection and Data Extraction

All identified records were imported into EndNote 2025 (version 2025.2; Clarivate, Philadelphia, PA, USA) for duplicate removal, and unique articles were subsequently uploaded to Rayyan (Rayyan Systems Inc., Cambridge, MA, USA; https://www.rayyan.ai, accessed on 2 May 2026) for study selection. Two reviewers (J.M.M. and C.D.A.) independently screened titles and abstracts, followed by full-text review of potentially eligible studies, based on predefined eligibility criteria. Disagreements were resolved by a third reviewer (C.E.S.). Data on study characteristics, patient demographics, and predefined outcomes were independently extracted by two reviewers (J.M.M. and C.D.A.) into a standardized form, with discrepancies resolved by a third reviewer (C.E.S.).

### 2.4. Study Endpoints

The primary outcomes were ACM and HFH at mid-term (6–12 months) and long-term (>12 months) follow-up. Secondary outcomes included functional recovery and procedural and safety outcomes. Functional outcomes at mid-term were analyzed separately at 6 and 12 months when data were available, comprising LVEF, change in LVEF from baseline, QRS duration, change in QRS duration, left ventricular end-systolic volume (LVESV), NYHA functional class, and 6 min walk test (6MWT) distance. Procedural and safety outcomes included pacing threshold—also analyzed at 6 and 12 months—procedure duration, fluoroscopy time, and periprocedural complications.

### 2.5. Risk of Bias Assessment

The methodological quality of each included RCT was independently assessed by two reviewers (J.M.M. and C.D.A.) using the Cochrane Risk of Bias tool version 2 (RoB2) [21]. Discrepancies were resolved by a third reviewer (C.E.S.). The certainty of evidence for each outcome was assessed using the Grading of Recommendations Assessment, Development and Evaluation (GRADE) framework [22].

### 2.6. Statistical Analysis

For dichotomous outcomes (ACM, HFH, periprocedural complications), pooled risk ratios (RRs) with 95% confidence intervals (CIs) were calculated. For continuous outcomes, mean differences (MDs) were used where studies reported the same measurement units; standardized mean differences (SMDs) were used for LVESV, given heterogeneity in measurement units across RCTs.

All meta-analyses were performed using random-effects models with restricted maximum likelihood estimation, in accordance with current Cochrane Handbook recommendations [23]. Statistical heterogeneity was quantified using the I^2^ statistic, interpreted per Cochrane thresholds: 0% to 40% (potentially unimportant), 30% to 60% (moderate), 50% to 90% (substantial), and 75% to 100% (considerable heterogeneity), in conjunction with the τ^2^ statistic and Cochran’s Q test (significance threshold *p* < 0.10) [23]. Prespecified subgroup analyses were conducted by CSP modality (LBBAP vs. HBP) for all outcomes, with formal testing of subgroup interactions reported. A leave-one-out sensitivity analysis was performed for each outcome to assess the influence of individual RCTs on pooled estimates. An additional sensitivity analysis excluding the four RCTs that permitted both LBBAP and HBP and were classified according to the predominant technique (>50% of implants) was performed to assess the influence of mixed CSP approaches. All analyses were performed in R version 4.5.2 (R Foundation for Statistical Computing, Vienna, Austria) using the “meta” package version 8.2.1, supplemented by TSA software version 0.9.5.10 Beta (Copenhagen Trial Unit, Copenhagen, Denmark) [24], to determine whether the cumulative evidence met the required information size for error-controlled conclusions.

## 3. Results

### 3.1. Search Results

**Figure 1:**
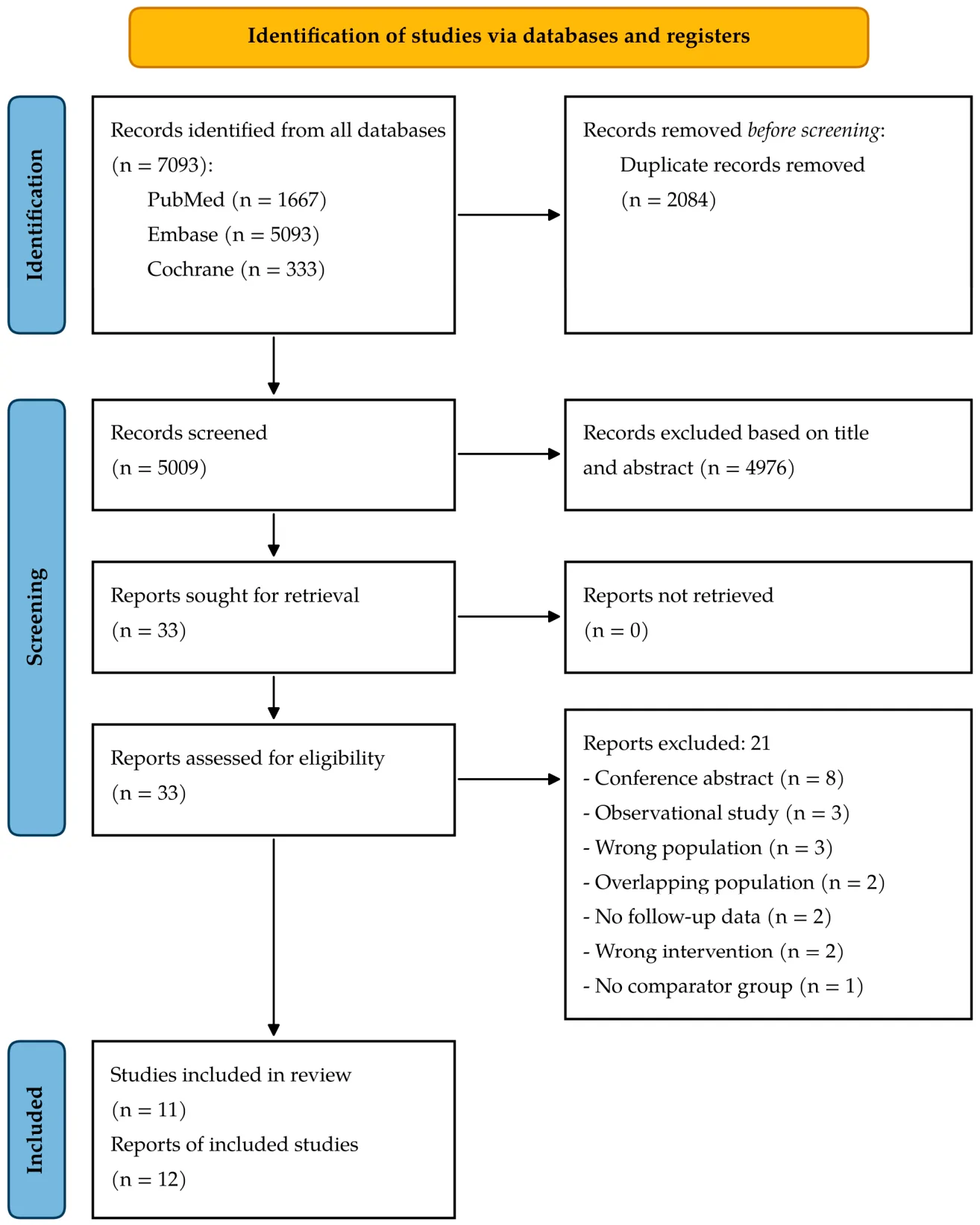
PRISMA flow diagram of study screening and selection.

A total of 7093 records were identified through database searches. Following the removal of duplicates, 5009 records underwent title and abstract screening, of which 4976 were excluded as irrelevant. Full-text review was performed on all remaining 33 records, of which 21 were excluded. The reasons for exclusions are detailed in Figure 1. The most frequent reason for exclusion was conference abstract (n = 8), followed by observational study (n = 3) and wrong population (n = 3). Ultimately, 11 randomized controlled trials (RCTs) comprising 12 reports were included in the final analysis (Figure 1).

### 3.2. Study Characteristics

The 11 included RCTs comprised 12 reports [13,14,15,16,17,19,25,26,27,28,29,30] and enrolled 1073 patients. Among the 10 parallel-arm RCTs [13,14,15,16,17,19,26,27,28,29], 523 patients were allocated to the CSP arm and 521 to the BiVP arm; 1 RCT from Lustgarten et al. [25] utilized a crossover design enrolling 29 patients. Studies were published between 2015 and 2026 across six countries (United States, Spain, Brazil, China, Slovenia, and Denmark). Most RCTs were conducted in a multicentric setting (n = 7) [15,16,17,19,25,26,29], with the remainder being single-center studies (n = 4) [13,14,27,28]. CSP modality was LBBAP in eight RCTs [13,14,15,16,17,19,28,29] and HBP in three RCTs [25,26,27]; four of the LBBAP RCTs permitted both LBBAP and HBP [14,16,28,29], with analysis performed according to the predominant technique. Of note, the report by Frandsen et al. [30] represents the long-term follow-up data of the RCT from Vinther et al. [27] and was analyzed accordingly. Follow-up duration ranged from 6 to 36 months.

Baseline characteristics were well-balanced across treatment arms in all included RCTs. The mean age ranged from 61 to 71 years, with a female proportion of 22% to 65% across RCTs. Mean baseline LVEF ranged from 27% to 30% across RCTs, and mean baseline QRS duration ranged from 162 to 184 ms. The proportion of nonischemic cardiomyopathy ranged from 33% to 100%, with substantial variability across RCTs. LBBB was present in most patients, with rates reaching 100% in 6 of the 10 parallel-arm RCTs [13,15,16,17,19,27]; the remaining RCTs reported LBBB rates ranging from 55% to 88% [14,25,26,28,29]. Mean baseline NYHA functional class ranged from 2.0 to 3.0 across studies.

Guideline-directed medical therapy use was variable across all included RCTs. Angiotensin-converting enzyme inhibitor/angiotensin receptor blocker/angiotensin receptor-neprilysin inhibitor was prescribed in 30% to 100% of patients, beta-blockers in 29% to 100%, and mineralocorticoid receptor antagonists in 20% to 100%. Sodium-glucose cotransporter-2 inhibitor use was reported in five RCTs [13,14,15,16,17], ranging from 29% to 94%, reflecting their increasing adoption during the study period. Detailed baseline characteristics of all included studies are presented in Table 1.

### 3.3. Primary Outcomes

#### 3.3.1. Clinical Events

##### All-Cause Mortality

**Figure 2:**
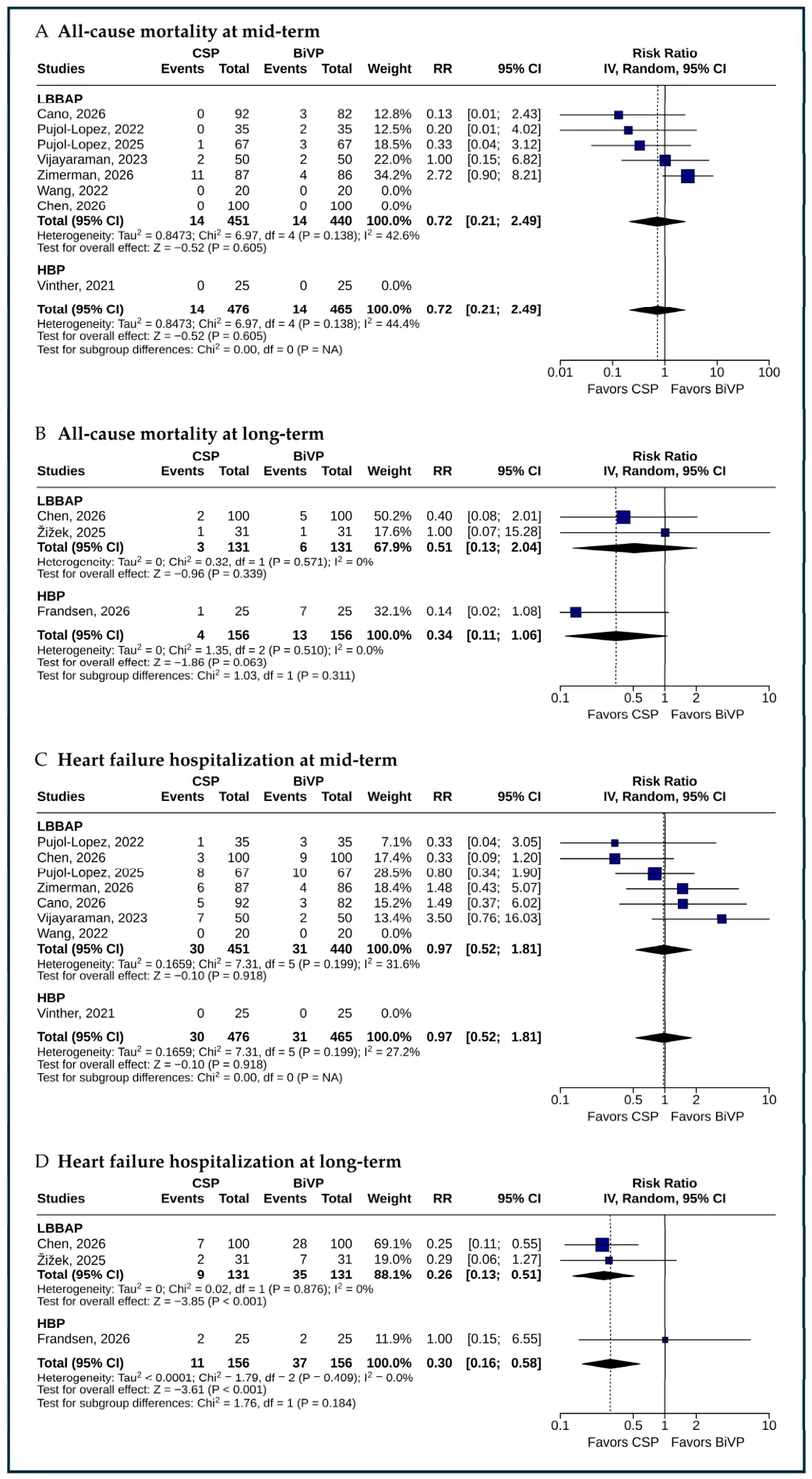
Clinical events. (**A**) All-cause mortality at mid-term (6–12 months). (**B**) All-cause mortality at long-term (follow-up > 12 months). (**C**) Heart failure hospitalization at mid-term. (**D**) Heart failure hospitalization at long-term. Included studies: [13,14,15,16,17,19,27,28,29,30]. Blue squares represent individual study estimates with corresponding 95% confidence intervals; black diamonds represent the pooled effect estimates with their 95% confidence intervals; the solid vertical line indicates the line of no effect; and the vertical dashed line indicates the overall pooled effect estimate. BiVP = biventricular pacing; CSP = conduction system pacing; HBP = His-bundle pacing; LBBAP = left bundle branch area pacing; NA = not applicable.

Data from eight RCTs (n = 941) revealed no significant difference in ACM at mid-term follow-up (6–12 months) between CSP and BiVP (RR 0.72; 95% CI 0.21 to 2.49; *p* = 0.605; I^2^ = 44.4%) (Figure 2A). At long-term follow-up (>12 months; range 22–60 months), three RCTs (n = 312) showed a trend toward ACM reduction with CSP (RR 0.34; 95% CI 0.11 to 1.06; *p* = 0.063; I^2^ = 0%) (Figure 2B).

##### Heart Failure Hospitalization

Among eight RCTs (n = 941), no significant difference in HFH was observed at mid-term (RR 0.97; 95% CI 0.52 to 1.81; *p* = 0.918; I^2^ = 27.2%) (Figure 2C). At long-term follow-up (>12 months; range 22–60 months), three RCTs (n = 312) demonstrated a significant decrease in HFH with CSP compared with BiVP (RR 0.30; 95% CI 0.16 to 0.58; *p* < 0.001; I^2^ = 0%) (Figure 2D).

### 3.4. Secondary Outcomes

#### 3.4.1. Functional Recovery

##### Left Ventricular Ejection Fraction

**Figure 3:**
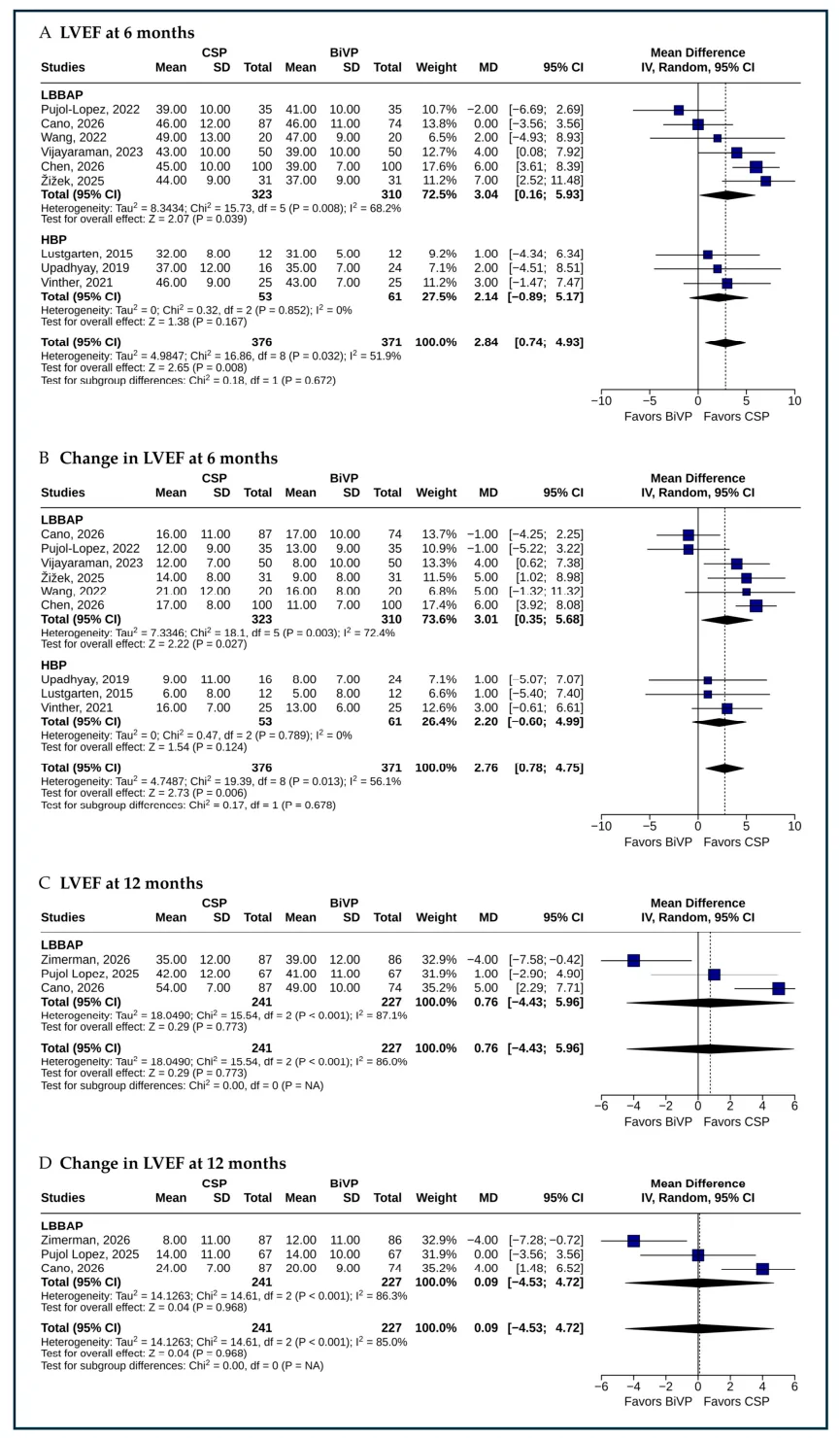
Functional outcomes. (**A**) LVEF at 6 months. (**B**) Change in LVEF at 6 months. (**C**) LVEF at 12 months. (**D**) Change in LVEF at 12 months. Included studies: [13,14,15,16,17,19,25,26,27,28,29]. Blue squares represent individual study estimates with corresponding 95% confidence intervals; black diamonds represent the pooled effect estimates with their 95% confidence intervals; the solid vertical line indicates the line of no effect; and the vertical dashed line indicates the overall pooled effect estimate. BiVP = biventricular pacing; CSP = conduction system pacing; HBP = His-bundle pacing; LBBAP = left bundle branch area pacing; LVEF = left ventricular ejection fraction; NA = not applicable.

At 6 months, data from nine RCTs (n = 747) demonstrated a significantly higher absolute LVEF with CSP compared with BiVP (MD 2.84%; 95% CI 0.74 to 4.93; *p* = 0.008; I^2^ = 51.9%) (Figure 3A), with a concordant significant improvement in LVEF from baseline (MD 2.76%; 95% CI 0.78 to 4.75; *p* = 0.006; I^2^ = 56.1%) (Figure 3B). At 12 months, data from three RCTs (n = 468) revealed no significant difference in either absolute LVEF (MD 0.76%; 95% CI −4.43 to 5.96; *p* = 0.773; I^2^ = 86.0%) (Figure 3C) or change from baseline (MD 0.09%; 95% CI −4.53 to 4.72; *p* = 0.968; I^2^ = 85.0%) (Figure 3D), with substantial heterogeneity across RCTs.

##### QRS Duration

**Figure 4:**
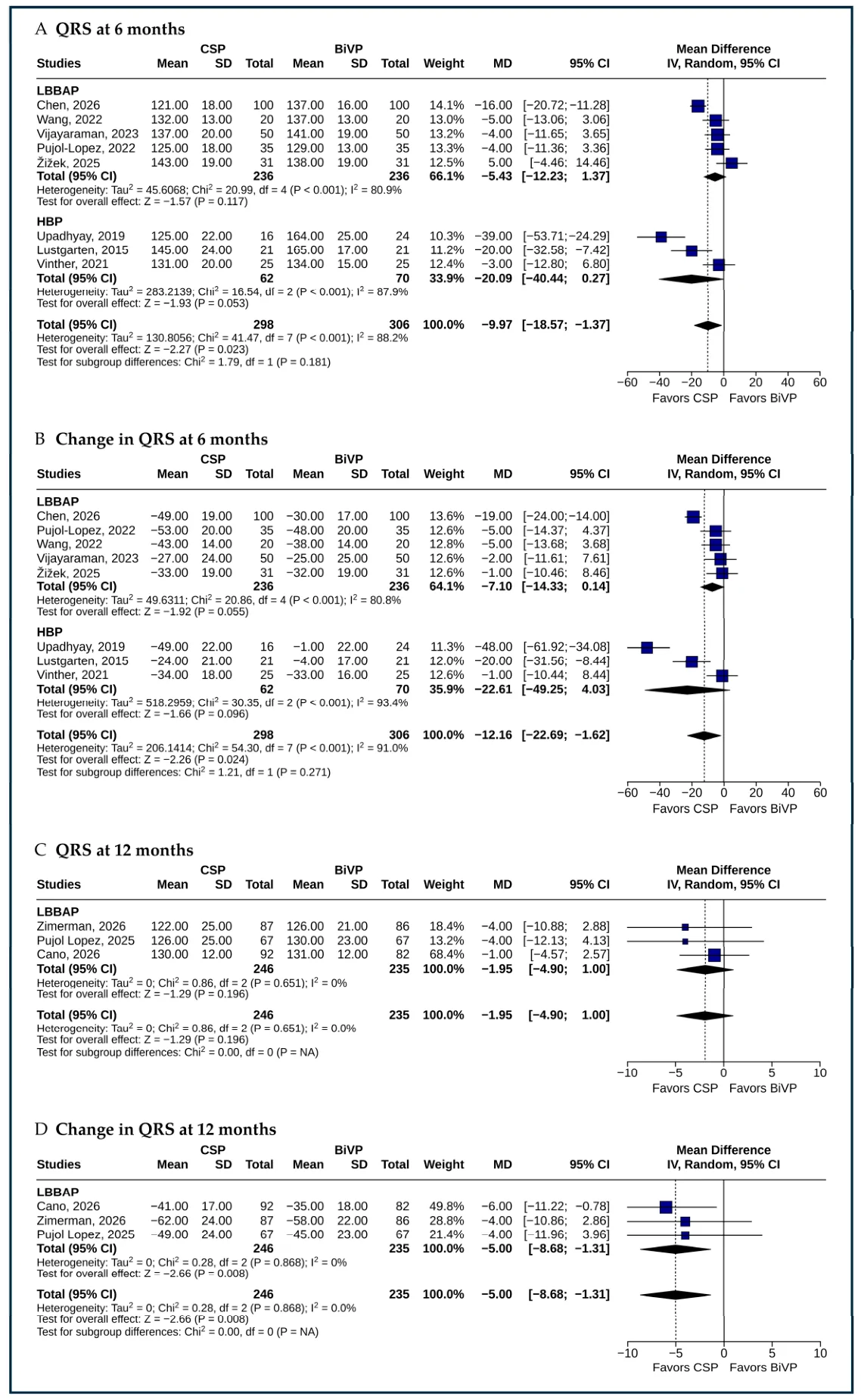
Functional outcomes. (**A**) QRS at 6 months. (**B**) Change in QRS at 6 months. (**C**) QRS at 12 months. (**D**) Change in QRS at 12 months. Included studies: [13,14,15,16,17,19,25,26,27,28,29]. Blue squares represent individual study estimates with corresponding 95% confidence intervals; black diamonds represent the pooled effect estimates with their 95% confidence intervals; the solid vertical line indicates the line of no effect; and the vertical dashed line indicates the overall pooled effect estimate. BiVP = biventricular pacing; CSP = conduction system pacing; HBP = His-bundle pacing; LBBAP = left bundle branch area pacing; NA = not applicable.

At 6 months, pooled data from eight RCTs (n = 604) showed a significantly shorter paced QRS duration with CSP versus BiVP (MD −9.97 ms; 95% CI −18.57 to −1.37; *p* = 0.023; I^2^ = 88.2%) (Figure 4A), with a concordant reduction from baseline (MD −12.16 ms; 95% CI −22.69 to −1.62; *p* = 0.024; I^2^ = 91.0%) (Figure 4B). At 12 months, three RCTs (n = 481) showed no significant difference in absolute QRS duration (MD −1.95 ms; 95% CI −4.90 to 1.00; *p* = 0.196; I^2^ = 0%) (Figure 4C), whereas the change in QRS from baseline remained significantly greater with CSP (MD −5.00 ms; 95% CI −8.68 to −1.31; *p* = 0.008; I^2^ = 0%) (Figure 4D).

##### Left Ventricular End-Systolic Volume

**Figure 5:**
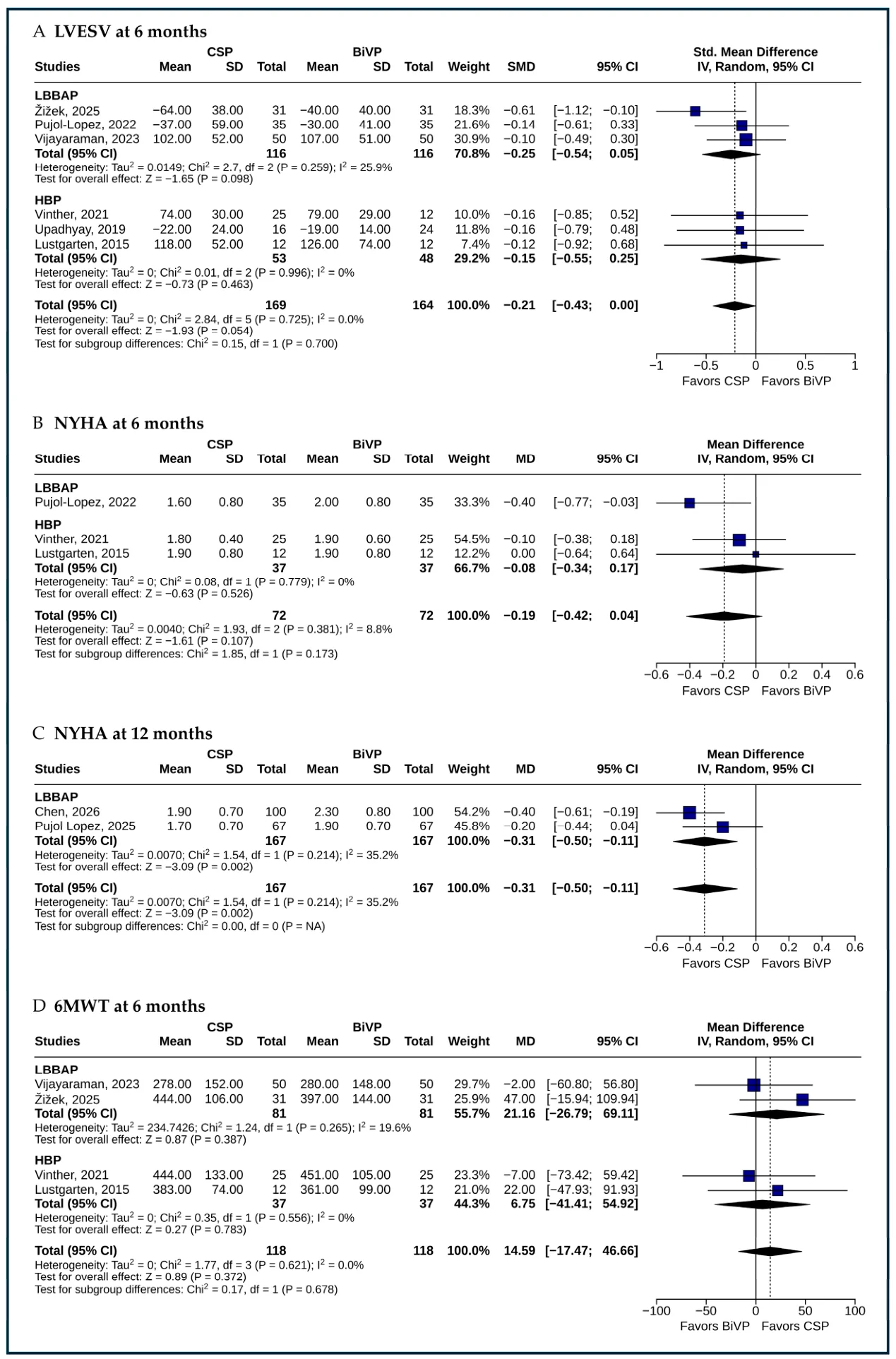
Functional outcomes. (**A**) LVESV at 6 months; (**B**) NYHA at 6 months; (**C**) NYHA at 12 months; (**D**) 6MWT at 6 months. Included studies: [13,14,15,25,26,27,28,29]. Blue squares represent individual study estimates with corresponding 95% confidence intervals; black diamonds represent the pooled effect estimates with their 95% confidence intervals; the solid vertical line indicates the line of no effect; and the vertical dashed line indicates the overall pooled effect estimate. BiVP = biventricular pacing; CSP = conduction system pacing; HBP = His-bundle pacing; LBBAP = left bundle branch area pacing; LVESV = left ventricular end-systolic volume; NA = not applicable; NYHA = New York Heart Association.

Among six RCTs (n = 333) reporting LVESV at 6 months, a trend toward lower LVESV with CSP compared with BiVP was observed (SMD −0.21; 95% CI −0.43 to 0.00; *p* = 0.054; I^2^ = 0%) (Figure 5A). At 12 months, only one RCT (n = 134) reported LVESV change, with no difference between CSP and BiVP (−28 ± 27 vs. −28 ± 28 mL) [14].

##### NYHA Functional Class

At 6 months, three RCTs (n = 144) showed no significant difference in NYHA class between CSP and BiVP (MD −0.19; 95% CI −0.42 to 0.04; *p* = 0.107; I^2^ = 8.8%) (Figure 5B). At 12 months, two RCTs (n = 334) demonstrated a significant improvement in NYHA functional class with CSP compared with BiVP (MD −0.31; 95% CI −0.50 to −0.11; *p* = 0.002; I^2^ = 35.2%) (Figure 5C).

##### Minute Walk Test

Among four RCTs (n = 236) reporting 6MWT at 6 months, no significant difference was observed between CSP and BiVP (MD 14.59 m; 95% CI −17.47 to 46.66; *p* = 0.372; I^2^ = 0%) (Figure 5D). At 12 months, a single RCT (n = 173) reported a numerically higher 6MWT distance with CSP compared with BiVP (390 ± 103 vs. 358 ± 100 m) [16].

#### 3.4.2. Procedural and Safety Outcomes

##### Pacing Threshold

**Figure 6:**
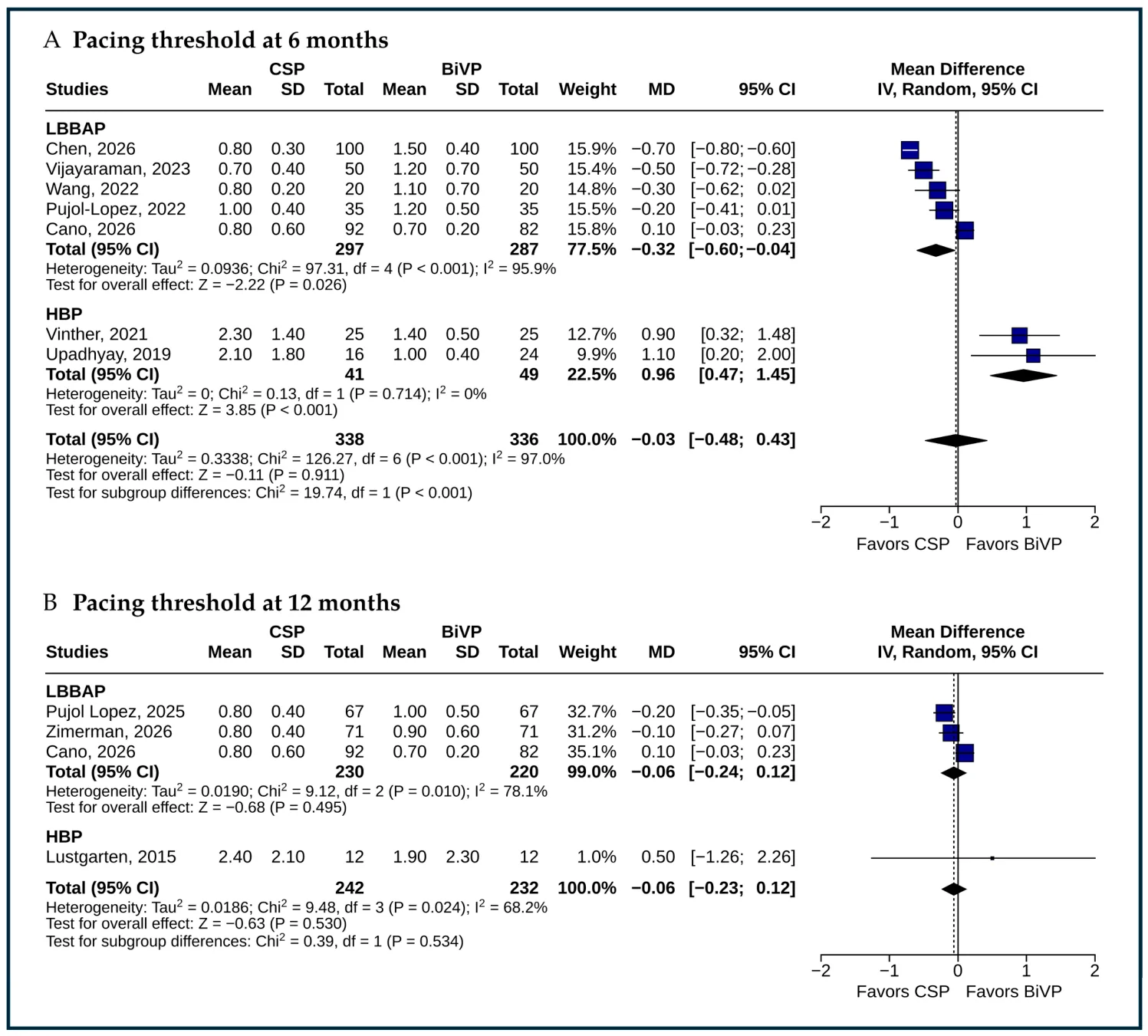
Procedural outcomes. (**A**) Pacing threshold at 6 months. (**B**) Pacing threshold at 12 months. Included studies: [14,15,16,17,19,25,26,27,28,29]. Blue squares represent individual study estimates with corresponding 95% confidence intervals; black diamonds represent the pooled effect estimates with their 95% confidence intervals; the solid vertical line indicates the line of no effect; and the vertical dashed line indicates the overall pooled effect estimate. BiVP = biventricular pacing; CSP = conduction system pacing; HBP = His-bundle pacing; LBBAP = left bundle branch area pacing.

At 6 months, seven RCTs (n = 674) revealed no significant overall difference in pacing threshold between CSP and BiVP (MD −0.03 V; 95% CI −0.48 to 0.43; *p* = 0.911; I^2^ = 97.0%) (Figure 6A). At 12 months, four RCTs (n = 474) similarly showed no significant difference (MD −0.06 V; 95% CI −0.23 to 0.12; *p* = 0.530; I^2^ = 68.2%) (Figure 6B).

##### Procedure Duration

**Figure 7:**
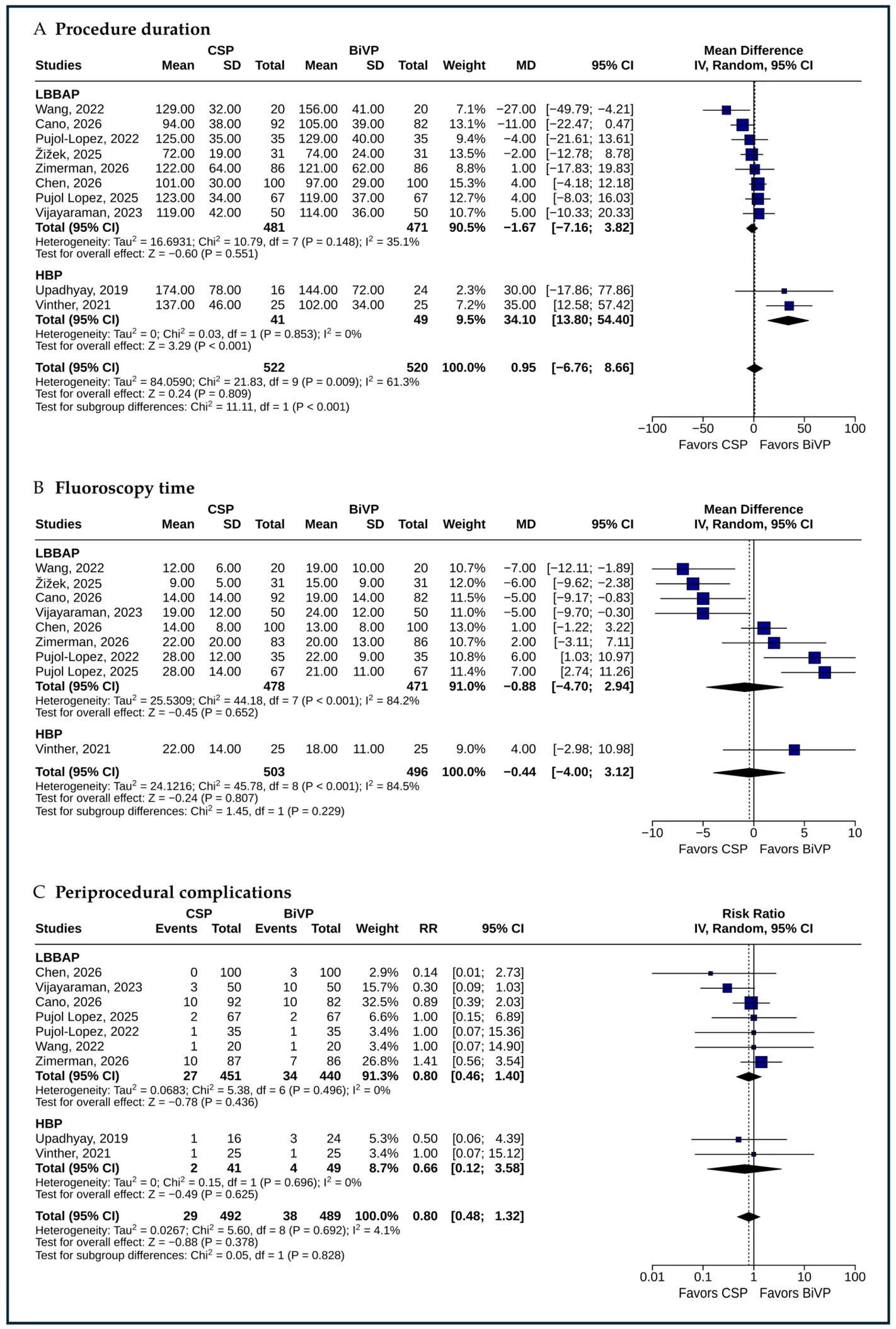
Procedural and safety outcomes. (**A**) Procedure duration. (**B**) Fluoroscopy time. (**C**) Periprocedural complications. Included studies: [13,14,15,16,17,19,26,27,28,29]. Blue squares represent individual study estimates with corresponding 95% confidence intervals; black diamonds represent the pooled effect estimates with their 95% confidence intervals; the solid vertical line indicates the line of no effect; and the vertical dashed line indicates the overall pooled effect estimate. BiVP = biventricular pacing; CSP = conduction system pacing; HBP = His-bundle pacing; LBBAP = left bundle branch area pacing.

Among 10 RCTs (n = 1042), no significant overall difference in procedure duration was observed between CSP and BiVP (MD 0.95 min; 95% CI −6.76 to 8.66; *p* = 0.809; I^2^ = 61.3%) (Figure 7A).

##### Fluoroscopy Time

Among nine RCTs (n = 999), fluoroscopy time did not differ significantly between CSP and BiVP (MD −0.44 min; 95% CI −4.00 to 3.12; *p* = 0.807; I^2^ = 84.5%) (Figure 7B).

##### Periprocedural Complications

Among nine RCTs (n = 981), the overall rate of periprocedural complications was comparable between CSP and BiVP (RR 0.80; 95% CI 0.48 to 1.32; *p* = 0.378; I^2^ = 4.1%) (Figure 7C).

### 3.5. Subgroup Analyses

Subgroup analyses by CSP modality revealed significant interactions for pacing threshold at 6 months and procedure duration (*p*-interaction < 0.001 for both): LBBAP was associated with lower thresholds versus BiVP, whereas HBP demonstrated higher thresholds (Figure 6A) and longer implantation time (Figure 7A). Both modalities provided comparable LVEF improvement, with no significant interactions for remaining functional or procedural outcomes. LBBAP was the main contributor to long-term HFH reduction, whereas HBP showed a numerically neutral result, though the subgroup interaction was not significant (*p* interaction = 0.184) (Figure 2D).

### 3.6. Sensitivity Analyses

Sensitivity analysis using a systematic leave-one-out approach demonstrated that pooled estimates were robust for most outcomes. Removal of Žižek et al. [13] changed the trend for ACM at long-term, which reached statistical significance (RR 0.27; 95% CI 0.08 to 0.95; *p* = 0.041). Secondary outcomes affected by leave-one-out exclusion are described in Supplementary Material, Table S2. Exclusion of the four RCTs that permitted both CSP techniques [14,16,28,29] did not materially alter the direction or statistical significance of the pooled estimates, with the exception of LVESV at 6 months, which reached statistical significance in favor of CSP (SMD −0.33; 95% CI −0.64 to −0.01; *p* = 0.043); long-term clinical outcomes were unchanged because none of these trials contributed to the long-term analyses (Supplementary Material, Table S3).

### 3.7. Quality of the Included Studies

Risk of bias was assessed using the Cochrane RoB2 tool. Overall, two of 11 RCTs were judged as low risk of bias and nine as some concerns, with no trial rated as high risk (Supplementary Material, Figure S1). Nine RCTs showed some concerns in the selection of the reported result, four in the randomization process, and one in deviations from the intended intervention. No RCT showed concerns about missing outcome data or the measurement of the outcome. Certainty of evidence per outcome, graded with GRADE, is reported in Supplementary Material, Table S4.

### 3.8. Trial Sequential Analysis

In the TSA of long-term HFH (Supplementary Material, Figure S2D), the cumulative Z-curve crossed the monitoring boundary, and the required information size (RIS) was attained, supporting the pooled effect favoring CSP, although this estimate was based on only three RCTs (n = 312). TSA for ACM at long-term did not reach the RIS; for ACM at mid-term and HFH at mid-term, RIS was not estimable (Supplementary Material, Figure S2A–C). Results for secondary outcomes are presented in Supplementary Material, Figures S3–S6.

## 4. Discussion

In this systematic review and meta-analysis of 11 RCTs and 1073 patients comparing CSP versus BiVP in HFrEF with wide QRS, in which LBBAP was the predominant CSP modality, the main findings with CSP include: a significant reduction in HFH, based on three RCTs and 312 patients, and a non-significant trend toward reduced ACM at long-term follow-up (>12 months), without a significant difference in either outcome at mid-term (6–12 months); greater LVEF improvement and QRS narrowing at 6 months, though the LVEF advantage was not sustained at 12 months; and sustained QRS reduction from baseline and significant NYHA functional class improvement at 12 months. Subgroup analysis by CSP modality revealed that at 6 months, LBBAP drove lower pacing thresholds versus BiVP, whereas HBP showed higher thresholds, with longer procedure duration at implant. The certainty of evidence was moderate for long-term HFH and low or very low for most other endpoints.

BiVP has been the evidence-based standard for CRT in HFrEF with LBBB for more than two decades, supported by Class I guideline recommendations [1,2]. However, non-physiological epicardial activation, a 30% non-response rate, and limitations in coronary sinus lead delivery provided the mechanistic and clinical rationale for CSP as a strategy to restore synchronized ventricular activation through the native His–Purkinje system [3,4,5,6,7,31], a premise directly evaluated in the present analysis.

The central clinical question is whether this physiological advantage of CSP translates into superior hard clinical outcomes. At mid-term follow-up, this remains uncertain as CSP was not associated with significant reductions in either ACM or HFH. These findings are consistent with Ferreira Felix et al. [12], who similarly found no significant difference in HFH (RR 1.02; 95% CI 0.21 to 4.90; *p* = 0.98; I^2^ = 46%) or ACM (RR 0.45; 95% CI 0.12 to 1.62; *p* = 0.219; I^2^ = 0%), although that analysis shares several trials with the present one. Importantly, these null findings should not be interpreted as evidence of equivalence. The relatively short follow-up and low event rates limited statistical power to detect differences in hard clinical outcomes, and the required information size could not be estimated by TSA for either endpoint. Thus, a clinically meaningful benefit or harm cannot be excluded. Moderate heterogeneity in ACM was driven largely by Zimerman et al. [16], in which CSP was associated with higher mortality than BiVP (Hazard ratio (HR) 3.36; 95% CI 1.05 to 10.71), accounting for 11 of 14 deaths in the pooled analysis. One potential contributor is operator experience, as 42.8% of CSP implants were performed by operators with fewer than 40 prior cases, substantially below the approximately 100–200 procedures associated with proficiency in the MELOS registry [32]. Whether operator experience explains the observed mortality signal remains uncertain, particularly as other contributing RCTs reported numerically fewer deaths with CSP [14,17,28]. These findings underscore the need for adequately powered trials with longer follow-up and standardized CSP expertise to determine whether its physiological advantage translates into durable clinical benefit.

In contrast, at long-term follow-up, CSP was associated with a 70% relative reduction in HFH, driven predominantly by LBBAP. However, this estimate derives from only three RCTs and 312 patients, approximately one-third of the mid-term dataset, and Chen et al. [15] accounted for approximately 69% of the pooled weight; the sustained reduction in HFH over 36 months reported in that trial (7.0% vs. 28.0%; HR 0.23; 95% CI 0.10 to 0.52; *p* < 0.001) is therefore the main contributor to the pooled estimate. Although TSA supported this result, large relative effects derived from few trials and events are prone to overestimation. Moreover, because different sets of trials contributed to each time horizon, the contrast between neutral mid-term and favorable long-term estimates may partly reflect between-study differences rather than a true time-dependent effect; progressive reverse remodeling and the accumulation of clinical events over longer follow-up are plausible explanations that require confirmation in adequately powered RCTs. A parallel trend toward ACM reduction was also observed at the same horizon, although the confidence interval was wide and the RIS was not reached (312 of 498 patients); this finding should therefore be regarded as hypothesis-generating. Prior meta-analyses showed similar findings regarding reductions in HFH [33,34,35] and ACM [35,36] with CSP but predominantly incorporated observational evidence, susceptible to selection bias and confounding, and lacked predefined temporal stratification, limiting direct comparability with the present RCT-restricted synthesis.

CSP produced significantly greater improvement in LVEF from baseline to 6 months, with no significant interaction according to CSP modality. This advantage was not sustained at 12 months; however, the 12-month estimate was derived from only three RCTs (n = 468), showed considerable heterogeneity (I^2^ = 86.0%), and was supported by very low-certainty evidence, limiting conclusions regarding durability of effect. The attenuation may be influenced in part by Zimerman et al. [16], in which LVEF improvement was significantly less pronounced with CSP than with BiVP (35% ± 12 vs. 39% ± 12). High periprocedural complication rates associated with stylet-driven leads in a population with advanced ventricular remodeling who are known to respond robustly to BiVP represent potential trial-level explanations, although this hypothesis cannot be formally tested given the limited number of studies. Conversely, Chen et al. [15] demonstrated sustained superiority of LBBAP in LVEF at 36 months (47.3% ± 10.6 vs. 41.5% ± 8.6; *p* < 0.001), while both Žižek et al. [13] (40% vs. 3%; *p* < 0.01) and Chen et al. [15] (65% vs. 44%; *p* < 0.001) reported higher super-response rates (LVEF ≥50% or absolute LVEF improvement ≥15%) with LBBAP. Together, these findings suggest that the apparent attenuation of benefit at 12 months may have been influenced by trial-level heterogeneity rather than necessarily reflecting loss of the physiological effect over time, although a true attenuation or chance cannot be excluded. A directional trend toward greater reduction in LVESV at 6 months further supported a potential early reverse remodeling effect, although the pooled estimate did not reach significance. Improvement in NYHA functional class was also non-significant at 6 months but became significant at 12 months, based on two LBBAP RCTs [14,15].

Electrical resynchronization followed a similarly nuanced pattern. At 6 months, CSP produced significantly shorter-paced QRS duration and a greater reduction from baseline compared with BiVP. At 12 months, the change in QRS from each patient’s own baseline remained significantly greater with CSP, suggesting a persistent resynchronization advantage [14,16,17], whereas absolute QRS duration did not differ significantly between arms, although the RIS was not attained for this endpoint. This divergence may reflect two converging factors: the three HBP trials that contributed the largest absolute QRS separations at 6 months [25,26,27] provided no 12-month absolute QRS data, and progressive BiVP device optimization may have narrowed the absolute gap without attenuating the between-group divergence from baseline. Similar QRS narrowing with CSP has been reported in prior meta-analyses, which predominantly included observational studies [33,36,37].

The modality-specific differences underlying these electrical findings were further reflected in procedural outcomes. HBP demonstrated feasibility in Upadhyay et al. [26] but failed to achieve significant clinical benefit in intention-to-treat analyses owing to a 48% crossover rate from implant difficulty, a pattern corroborated by Vinther et al. [27]. These constraints drove the adoption of LBBAP, which accounted for 8 of the 11 included RCTs and offered more reliable septal capture with lower and more stable thresholds [11,38]. At the pooled level, no significant overall difference in procedure duration or fluoroscopy time was observed between CSP and BiVP, possibly reflecting variability in operator learning curves, although heterogeneity was substantial and the certainty of evidence was very low. However, a significant modality interaction was identified for pacing threshold at 6 months and procedure duration: LBBAP was associated with significantly lower pacing thresholds than BiVP, whereas HBP demonstrated significantly higher thresholds and longer implantation time, consistent with the established technical demands of His-bundle capture [26,27]. Elevated pacing thresholds carry direct implications for device longevity and risk of loss of capture [39]. Periprocedural complication rates were comparable between CSP and BiVP, suggesting an acceptable safety profile in experienced operators, although the RIS was not reached and the certainty of evidence was low. Taken together, these findings are driven predominantly by LBBAP, as randomized evidence for HBP is limited to three small trials.

### Strengths and Limitations

This analysis has several limitations. First, despite representing the largest pooled RCT analysis of CSP versus BiVP to date, the overall sample remains modest relative to the RIS for definitive conclusions on hard clinical outcomes, a boundary explicitly characterized by prespecified TSA. Second, heterogeneity in CSP definition across trials, including variability in LBBAP implantation criteria, left bundle branch capture confirmation methodology, definitions of CSP success, and operator experience, may have attenuated pooled estimates and affected their comparability, partially addressed through prespecified subgroup analysis by CSP modality. Third, moderate to substantial heterogeneity was identified in some outcomes, partly attributable to high crossover rates between arms, which differed substantially across trials (up to 48% from HBP to BiVP in Upadhyay et al. [26]) and were explored through prespecified leave-one-out sensitivity analyses and subgroup analyses by CSP modality. Intention-to-treat data were preferred whenever available; for Upadhyay et al. [26], whose full report presented results only by treatment received, these data were used, which may favor HBP, although exclusion of this trial did not materially change any pooled estimate. Under intention-to-treat analysis, crossover would be expected to dilute between-group differences toward the null. In addition, pre-crossover data were not reported for the crossover trial by Lustgarten et al. [25]; data from both pacing periods were therefore analyzed as independent groups, a conservative approach that assigns less weight to this trial. Fourth, variability in follow-up duration across contributing RCTs challenges the synthesis of time-varying outcomes, addressed through predefined separate analyses at 6 and 12 months and a distinct mid-term versus long-term stratification for clinical events; however, different sets of trials contributed to each time horizon, limiting comparisons across horizons. Fifth, several endpoints were informed by only two to four RCTs, which reduces the statistical power of subgroup interaction tests and the precision of heterogeneity estimates. Sixth, LBBAP was the predominant modality in 8 of the 11 RCTs; therefore, the pooled CSP estimates mainly reflect LBBAP, and the evidence for HBP is limited. Finally, nine of the 11 RCTs were judged to have some concerns in the RoB2 assessment, and the certainty of evidence was low or very low for several clinically relevant endpoints.

## 5. Conclusions

In patients with HFrEF and wide QRS, CSP may reduce long-term HFH compared with BiVP, supported by moderate-certainty evidence, while the observed trend toward lower long-term ACM remains non-significant and hypothesis-generating. These long-term findings should be interpreted cautiously, as they are derived from only three RCTs comprising 312 patients. Mid-term clinical outcomes did not differ significantly between strategies. Greater LVEF improvement and QRS narrowing at 6 months and significant improvement in NYHA class at 12 months further support CSP as a viable alternative to conventional BiVP for CRT. However, because randomized evidence predominantly reflects LBBAP, these findings should not be extrapolated to HBP. Larger, adequately powered RCTs with longer follow-up are needed to determine whether the physiological and remodeling advantages of CSP translate into durable reductions in HFH and mortality and to strengthen the certainty of the evidence.

## Figures and Tables

**Table 1:** Baseline characteristics of included studies.

	CSP/BiVP												
Study	Country	Setting	CSP Modality	Patients, n	Female, %	Age, y	Baseline LVEF, %	NICM, %	Baseline NYHA *	Baseline QRS, ms	LBBB, %	Follow-Up (mo) *	GDMT, %
Cano 2026 [17]	Spain	Multicentric	LBBAP	92/83	34/33	67 ± 10/69 ± 12	30 ± 8/29 ± 7	73/72	2.0/2.2	171 ± 20/166 ± 21	100	12	ACEI/ARB/ARNI: 94/93 BB: 86/86 MRA: 77/72 SGLT2i: 90/84
Zimerman 2026 [16]	Brazil	Multicentric	LBBAP †	87/86	52/48	61 ± 9/63 ± 10	27 ± 6/27 ± 8	85/90	2.4/2.5	184 ± 23/184 ± 23	100	12	ACEI/ARB/ARNI: 93/90 BB: 91/88 MRA: 78/88 SGLT2i: 29/41
Chen 2026 [15]	China	Multicentric	LBBAP	100/100	33/31	64 ± 10/65 ± 10	28 ± 4/28 ± 5	84/81	2.9/3.0	170 ± 19/167 ± 18	100	36	ACEI/ARB/ARNI: 97/98 BB: 96/95 MRA: 94/92 SGLT2i: 41/43
Pujol-Lopez 2025 [14]	Spain	Single	LBBAP†	67/67	28/25	69 ± 9/69 ± 9	28 ± 6/27 ± 7	66/66	2.3/2.4	175 ± 22/175 ± 23	55/57	12	ACEI/ARB/ARNI: 90/87 BB: 81/87 MRA: 73/67 SGLT2i: 88/88
Žižek 2025 [13]	Slovenia	Single	LBBAP	31/31	26/32	65 ± 10/68 ± 16	30 ± 5/28 ± 6	65/71	2.2/2.3	176 ± 18/170 ± 19	100	22	ACEI/ARB/ARNI: 100/97 BB: 100/97 MRA: 100/97 SGLT2i: 94/94
Vijayaraman 2023 [29]	USA	Multicentric	LBBAP †	50/50	22/40	71 ± 13/69 ± 12	30 ± 9/31 ± 9	60/62	2.5/2.5	164 ± 26/166 ± 28	62/62	6	ACEI/ARB/ARNI: 88/64 BB: 90/94 MRA: 28/30 SGLT2i: NA
Pujol-Lopez 2022 [28]	Spain	Single	LBBAP †	35/35	34/29	66 ± 9/68 ± 9	27 ± 7/28 ± 7	69/69	2.4/2.4	177 ± 21/178 ± 22	60/63	6	ACEI/ARB/ARNI: 31/30 BB: 29/31 MRA: 25/20 SGLT2i: NA
Wang 2022 [19]	China	Multicentric	LBBAP	20/20	65/35	62 ± 11/65 ± 11	28 ± 5/31 ± 6	100/100	2.4/2.5	175 ± 14/175 ± 14	100	6	ACEI/ARB/ARNI: 90/95 BB: 95/95 MRA: 95/90 SGLT2i: NA
Vinther 2021 [27]	Denmark	Single	HBP	25/25	44/28	64 ± 9/68 ± 9	30 ± 6/30 ± 8	80/76	2.4/2.4	165 ± 14/167 ± 16	100	6	ACEI/ARB/ARNI: 88/96 BB: 80/92 MRA: 64/72 SGLT2i: NA
Upadhyay 2019 [26]	USA	Multicentric	HBP	16/24	44/33	63 ± 13/66 ± 12	28 ± 9/27 ± 6	38/33	3.0/2.8	174 ± 18/165 ± 17	88	12	ACEI/ARB/ARNI: 75/75 BB: 94/100 MRA: 44/29 SGLT2i: NA
Lustgarten 2015 ‡ [25]	USA	Multicentric	HBP	29	35	71 ± 24	27 ± 9	45	2.8	162 ± 45	97	6	NA

Values are mean ± SD unless otherwise indicated. * Mean or median. † Studies using both CSP modalities were classified and analyzed according to the predominant technique. ‡ Crossover design; total N shown. ACEI = angiotensin-converting enzyme inhibitor; ARB = angiotensin receptor blocker; ARNI = angiotensin receptor-neprilysin inhibitor; BB = beta-blocker; BiVP = biventricular pacing; CSP = conduction system pacing; GDMT = guideline-directed medical therapy; HBP = His-bundle pacing; LBBAP = left bundle branch area pacing; LBBB = left bundle branch block; LVEF = left ventricular ejection fraction; MRA = mineralocorticoid receptor antagonist; NA = not available; NICM = nonischemic cardiomyopathy; NYHA = New York Heart Association; SGLT2i = sodium-glucose cotransporter-2 inhibitor.

## Data Availability

The data used for this manuscript are publicly available, as this is a systematic review. The corresponding author will respond to inquiries regarding synthesized datasets upon reasonable request.

## Supplementary Materials

The following supporting information can be downloaded at: https://www.mdpi.com/article/10.3390/jcm15208087/s1, Table S1: Search strategies in (A) MEDLINE, (B) EMBASE, and (C) Cochrane Central Register of Controlled Trials (CENTRAL); Table S2: Sensitivity analysis: outcomes affected by leave-one-out exclusion; Table S3: Sensitivity analysis excluding the four RCTs that permitted both CSP techniques (Pujol-Lopez 2022 [28], Vijayaraman 2023 [29], Pujol-Lopez 2025 [14], and Zimerman 2026 [16]); Figure S1: Risk of Bias Assessments According to Risk of Bias 2 of the Included Studies; Table S4: Grading of Recommendations Assessment, Development, and Evaluation (GRADE) Summary of Findings; Figure S2: Trial sequential analysis of effects of CSP versus BiVP on (A) all-cause mortality (ACM) at mid-term, (B) ACM at long-term, (C) heart failure hospitalization (HFH) at mid-term, and (D) HFH at long-term; Figure S3: Trial sequential analysis of effects of CSP versus BiVP on (A) left ventricular ejection fraction (LVEF) at 6 months, (B) change in LVEF at 6 months, (C) LVEF at 12 months, and (D) change in LVEF at 12 months; Figure S4: Trial sequential analysis of effects of CSP versus BiVP on (A) QRS at 6 months, (B) change in QRS at 6 months, (C) QRS at 12 months, and (D) change in QRS at 12 months; Figure S5: Trial sequential analysis of effects of CSP versus BiVP on (A) New York Heart Association (NYHA) class at 6 months, (B) NYHA class at 12 months, (C) 6-minute walk test (6MWT) distance at 6 months, and (D) pacing threshold at 6 months; Figure S6: Trial sequential analysis of effects of CSP versus BiVP on (A) pacing threshold at 12 months, (B) procedure duration, (C) fluoroscopy time, and (D) procedural complications; Figure S7: PRISMA 2020 checklist. References [13,14,15,16,17,19,25,26,27,28,29,30] are cited in the Supplementary Materials.
