# Supplementary material for: Mid-Term and Long-Term Clinical Events with Conduction System Pacing Versus Biventricular Pacing in HFrEF with Wide QRS: A Systematic Review and Meta-Analysis of Randomized Controlled Trials with Trial Sequential Analysis
Source: J Clin Med. 2026 Oct 18;15(20):8087. doi: 10.3390/jcm15208087 (PMC13705894; doi:10.3390/jcm15208087)
Supplement: Supplementary file 1 [file jcm-15-08087-s001.zip › jcm-4532564-supplementary.pdf]

**Supplementary material**

**Supplemental Table S1:** Search strategies in (A) MEDLINE, (B) EMBASE, and (C) Cochrane Central Register of Controlled Trials (CENTRAL)

A

| Database: MEDLINE from inception to April15, 2026 | |
|---|---|
| Search strategy | Results |
| ("heart failure"[MeSH Terms] OR "heart failure" OR HF) AND ("Cardiac Resynchronization Therapy"[MeSH Terms] OR "cardiac resynchronization therapy" OR CRT OR "Biventricular pacing" OR "BiVP") AND ("conduction system pacing" OR CSP OR "His bundle" OR HBP OR "His Pacing" OR "His CRT" OR "Left Bundle" OR LBBP OR LBBAP) | 1,667 |

B

| Database: EMBASE from inception to April15, 2026 | |
|---|---|
| Search strategy | Results |
| ('heart failure'/exp OR 'heart failure' OR 'hf') AND ('cardiac resynchronization therapy'/exp OR 'cardiac resynchronization therapy' OR 'crt' OR 'biventricular pacing' OR 'bivp') AND ('conduction system pacing' OR 'csp' OR 'his bundle' OR 'hbp' OR 'his pacing' OR 'his crt' OR 'left bundle' OR 'lbbp' OR 'lbbap') | 5,093 |

C

| Database: Cochrane Central Register of Controlled Trials (CENTRAL) from inception to April15, 2026 | |
|---|---|
| Search strategy | Results |
| ("heart failure" OR HF) AND ("cardiac resynchronization therapy" OR CRT OR "biventricular pacing" OR "BiVP") AND ("conduction system pacing" OR CSP OR "His bundle" OR HBP OR "His Pacing" OR "His CRT" OR "Left Bundle" OR LBBP OR LBBAP) | 333 |

**Supplemental Table S2.** Sensitivity analysis: outcomes affected by leave-one-out exclusion

| Outcome | Study omitted | Trials remaining (n) | Sample size | Effect estimate (95% CI) | I² |
|---|---|---|---|---|---|
| ACM at long-term | Žižek, 2025 [13] | 2 | 250 | RR 0.27; 95% CI 0.08 to 0.95; p = 0.041 | 0% |
| QRS at 6 months | Chen, 2026 [15] | 7 | 404 | MD −9.1 ms; 95% CI −19.04 to 0.85; p = 0.073 | 80.2% |
| QRS at 6 months | Lustgarten, 2015 [25] | 7 | 562 | MD −8.73 ms; 95% CI −18.10 to 0.63; p = 0.068 | 84.5% |
| Change in QRS at 6 months | Chen, 2026 [15] | 7 | 404 | MD −11.16 ms; 95% CI −23.30 to 0.98; p = 0.072 | 85.9% |
| Change in QRS at 6 months | Lustgarten, 2015 [25] | 7 | 562 | MD −11.14 ms; 95% CI −23.06 to 0.79; p = 0.067 | 88.5% |
| Change in QRS at 12 months | Cano, 2026 [17] | 2 | 307 | MD −4.00 ms; 95% CI −9.20 to 1.20; p = 0.131 | 0% |
| Pacing threshold at 12 months | Cano, 2026 [17] | 3 | 300 | MD −0.15 V; 95% CI −0.26 to -0.04; p = 0.008 | 0% |

ACM, all-cause mortality; CI, confidence interval; MD, mean difference; RR, risk ratio.

**Supplemental Table S3.** Sensitivity analysis excluding the four RCTs that permitted both CSP techniques (Pujol-Lopez 2022 [28], Vijayaraman 2023 [29], Pujol-Lopez 2025 [14], and Zimerman 2026 [16])

| Outcome | Trials remaining (n) | Sample size | Effect estimate (95% CI) | I² | Change vs. primary analysis |
|---|---|---|---|---|---|
| ACM at mid-term | 4 | 464 | RR 0.13; 95% CI 0.01 to 2.43; p = 0.171* | NA | None |
| HFH at mid-term | 4 | 464 | RR 0.68; 95% CI 0.16 to 2.95; p = 0.611 | 58.1% | None |
| ACM at long-term | 3 | 312 | Unchanged† | — | None |
| HFH at long-term | 3 | 312 | Unchanged† | — | None |
| LVEF at 6 months | 7 | 577 | MD 3.34%; 95% CI 1.04 to 5.64; p = 0.004 | 47.7% | None |
| Change in LVEF at 6 months | 7 | 577 | MD 3.08%; 95% CI 0.77 to 5.39; p = 0.009 | 56.4% | None |
| LVEF at 12 months | 1 | 161 | NE | — | NE |
| Change in LVEF at 12 months | 1 | 161 | NE | — | NE |
| QRS at 6 months | 6 | 434 | MD −12.33 ms; 95% CI −24.08 to −0.58; p = 0.040 | 90.5% | None |

| | | | | | |
|---|---|---|---|---|---|
| Change in QRS at 6 months | 6 | 434 | MD −15.19 ms; 95% CI −28.92 to −1.46; p = 0.030 | 93.0% | None |
| QRS at 12 months | 1 | 174 | NE | — | NE |
| Change in QRS at 12 months | 1 | 174 | NE | — | NE |
| LVESV at 6 months | 4 | 163 | SMD −0.33; 95% CI −0.64 to −0.01; p = 0.043 | 0% | Reached significance |
| NYHA class at 6 months | 2 | 74 | MD −0.08; 95% CI −0.34 to 0.17; p = 0.526 | 0% | None |
| NYHA class at 12 months | 1 | 200 | NE | — | NE |
| 6MWT at 6 months | 3 | 136 | MD 21.62 m; 95% CI −16.63 to 59.86; p = 0.268 | 0% | None |
| Pacing threshold at 6 months | 5 | 504 | MD 0.15 V; 95% CI −0.50 to 0.79; p = 0.660 | 97.8% | None |
| Pacing threshold at 12 months | 2 | 198 | MD 0.10 V; 95% CI −0.03 to 0.23; p = 0.123 | 0% | None |
| Procedure duration | 6 | 566 | MD 1.89 min; 95% CI −14.81 to 18.59; p = 0.824 | 86.5% | None |
| Fluoroscopy time | 5 | 526 | MD −2.80 min; 95% CI −6.70 to 1.10; p = 0.159 | 78.8% | None |
| Periprocedural complications | 5 | 504 | RR 0.77; 95% CI 0.38 to 1.54; p = 0.462 | 0% | None |

*Only one trial (Cano 2026 [17]) contributed events; the remaining trials reported no events in either arm. †None of the excluded trials contributed to long-term clinical events. 6MWT, 6-minute walk test; ACM, all-cause mortality; CI, confidence interval; CSP, conduction system pacing; HFH, heart failure hospitalization; LVEF, left ventricular ejection fraction; LVESV, left ventricular end-systolic volume; MD, mean difference; NA, not applicable; NE, not estimable (fewer than two trials remaining); NYHA, New York Heart Association; RR, risk ratio; SMD, standardized mean difference.

**Supplemental Figure S1:** Risk of Bias Assessments According to Risk of Bias 2 of the Included Studies. Included studies: [13–17,19,25–30].

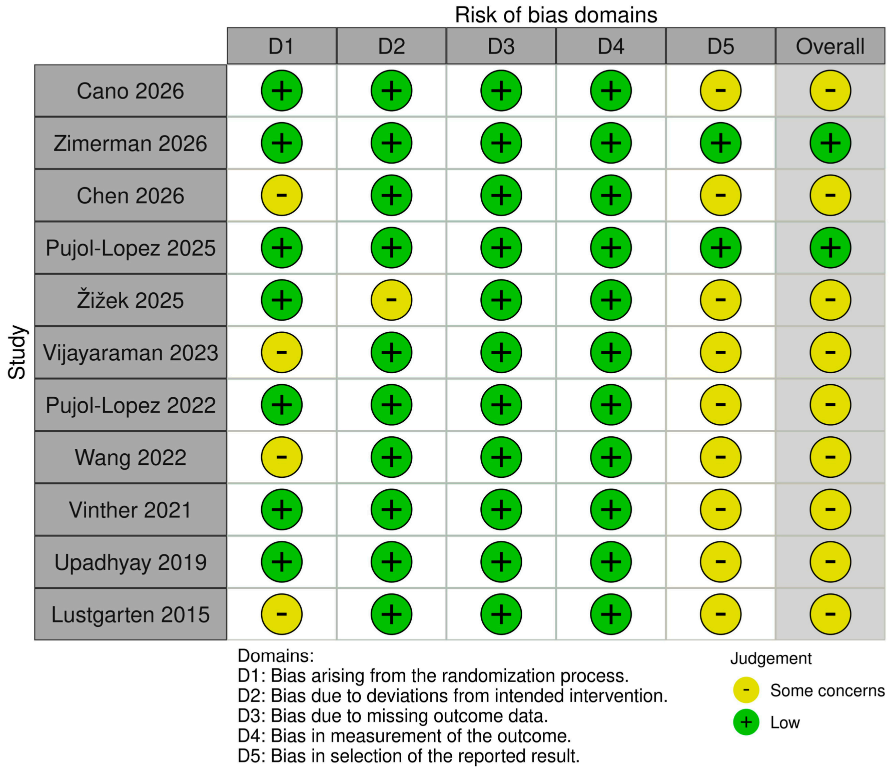

**Supplemental Table S4:** Grading of Recommendations Assessment, Development, and Evaluation (GRADE) Summary of Findings

| Outcomes | **Anticipated absolute effects*** (95% CI) | | Relative effect (95% CI) | № of participants (studies) | Certainty of the evidence (GRADE) |
|---|---|---|---|---|---|
| | **Risk with BiVP** | **Risk with CSP** | | | |
| All-cause mortality at mid-term (6-12 months) | 30 per 1000 | **22 per 1000** (6 to 75) | **RR 0.72** (0.21 to 2.49) | 941 (8 RCTs) | ⊕⊕◯◯ Low[a,b] |
| All-cause mortality at long-term (>12 months) | 83 per 1000 | **28 per 1000** (9 to 88) | **RR 0.34** (0.11 to 1.06) | 312 (3 RCTs) | ⊕⊕◯◯ Low[a,b] |
| Heart failure hospitalization at mid-term (6-12 months) | 67 per 1000 | **65 per 1000** (35 to 121) | **RR 0.97** (0.52 to 1.81) | 941 (8 RCTs) | ⊕⊕◯◯ Low[a,b] |
| Heart failure hospitalization at long-term (>12 months) | 237 per 1000 | **71 per 1000** (38 to 138) | **RR 0.30** (0.16 to 0.58) | 312 (3 RCTs) | ⊕⊕⊕◯ Moderate[a] |
| LVEF at 6 months | The mean LVEF ranged from **31 to 47** % | MD **2.84 % higher** (0.74 higher to 4.93 higher) | - | 747 (9 RCTs) | ⊕⊕◯◯ Low[a,c] |
| Change in LVEF at 6 months | The mean change in LVEF ranged from **5 to 17** % | MD **2.76 % higher** (0.78 higher to 4.75 higher) | - | 747 (9 RCTs) | ⊕⊕◯◯ Low[a,d] |
| LVEF at 12 months | The mean LVEF ranged from **39 to 49** % | MD **0.76 % higher** (4.43 lower to 5.96 higher) | - | 468 (3 RCTs) | ⊕◯◯◯ Very low[a,b,e] |
| Change in LVEF at 12 months | The mean change in LVEF ranged from **12 to 20** % | MD **0.09 % higher** (4.53 lower to 4.72 higher) | - | 468 (3 RCTs) | ⊕◯◯◯ Very low[a,b,f] |

| | | | | | |
|---|---|---|---|---|---|
| QRS duration at 6 months | The mean QRS duration ranged from **129 to 165** ms | MD **9.97 ms lower** (18.57 lower to 1.37 lower) | - | 604 (8 RCTs) | ⊕⊕◯◯ Low[a,g] |
| Change in QRS duration at 6 months | The mean change in QRS duration ranged from **-48 to -1** ms | MD **12.16 ms lower** (22.69 lower to 1.62 lower) | - | 604 (8 RCTs) | ⊕⊕◯◯ Low[a,h] |
| QRS duration at 12 months | The mean QRS duration ranged from **126 to 131** ms | MD **1.95 ms lower** (4.90 lower to 1.00 higher) | - | 481 (3 RCTs) | ⊕⊕◯◯ Low[a,b] |
| Change in QRS duration at 12 months | The mean change in QRS duration ranged from **-58 to -35** ms | MD **5.00 ms lower** (8.68 lower to 1.31 lower) | - | 481 (3 RCTs) | ⊕⊕⊕◯ Moderate[a] |
| LVESV at 6 months | The mean LVESV ranged from **-40 to 126** | SMD **0.21 SD lower** (0.43 lower to 0) | - | 333 (6 RCTs) | ⊕⊕◯◯ Low[a,b] |
| NYHA class at 6 months | The mean NYHA class ranged from **1.9 to 2.0** | MD **0.19 lower** (0.42 lower to 0.04 higher) | - | 144 (3 RCTs) | ⊕⊕◯◯ Low[a,b] |
| NYHA class at 12 months | The mean NYHA class ranged from **1.9 to 2.3** | MD **0.31 lower** (0.50 lower to 0.11 lower) | - | 334 (2 RCTs) | ⊕⊕⊕◯ Moderate[a] |
| 6MWT at 6 months | The mean 6MWT ranged from **280 to 451** m | MD **14.59 m higher** (17.47 lower to 46.66 higher) | - | 236 (4 RCTs) | ⊕⊕◯◯ Low[a,b] |
| Pacing threshold at 6 months | The mean pacing threshold ranged from **0.7 to 1.5** V | MD **0.03 V lower** (0.48 lower to 0.43 higher) | - | 674 (7 RCTs) | ⊕◯◯◯ Very low[a,b,i] |

| | | | | | |
|---|---|---|---|---|---|
| Pacing threshold at 12 months | The mean pacing threshold ranged from **0.7 to 1.9** V | MD **0.06 V lower** (0.23 lower to 0.12 higher) | - | 474 (4 RCTs) | ⊕◯◯◯ Very low[a,b,j] |
| Procedure duration | The mean procedure duration ranged from **74 to 156** min | MD **0.95 min higher** (6.76 lower to 8.66 higher) | - | 1042 (10 RCTs) | ⊕◯◯◯ Very low[a,b,k] |
| Fluoroscopy time | The mean fluoroscopy time ranged from **13 to 24** min | MD **0.44 min lower** (4.00 lower to 3.12 higher) | - | 999 (9 RCTs) | ⊕◯◯◯ Very low[a,b,l] |
| Periprocedural complications | 78 per 1000 | **62 per 1000** (37 to 103) | **RR 0.80** (0.48 to 1.32) | 981 (9 RCTs) | ⊕⊕◯◯ Low[a,b] |

Explanations:

a. Risk of bias was rated as 'some concerns' for most trials (9 of 11), based on the Cochrane RoB2 assessment

b. The effect estimate was imprecise, with a 95% confidence interval including no effect

c. Substantial heterogeneity was observed across studies ($I^2$ = 51.9%)

d. Substantial heterogeneity was observed across studies ($I^2$ = 56.1%)

e. Substantial heterogeneity was observed across studies ($I^2$ = 86.0%)

f. Substantial heterogeneity was observed across studies ($I^2$ = 85.0%)

g. Substantial heterogeneity was observed across studies ($I^2$ = 88.2%)

h. Substantial heterogeneity was observed across studies ($I^2$ = 91.0%)

i. Substantial heterogeneity was observed across studies ($I^2$ = 97.0%)

j. Substantial heterogeneity was observed across studies ($I^2$ = 68.2%)

k. Substantial heterogeneity was observed across studies ($I^2$ = 61.3%)

l. Substantial heterogeneity was observed across studies ($I^2$ = 84.5%)

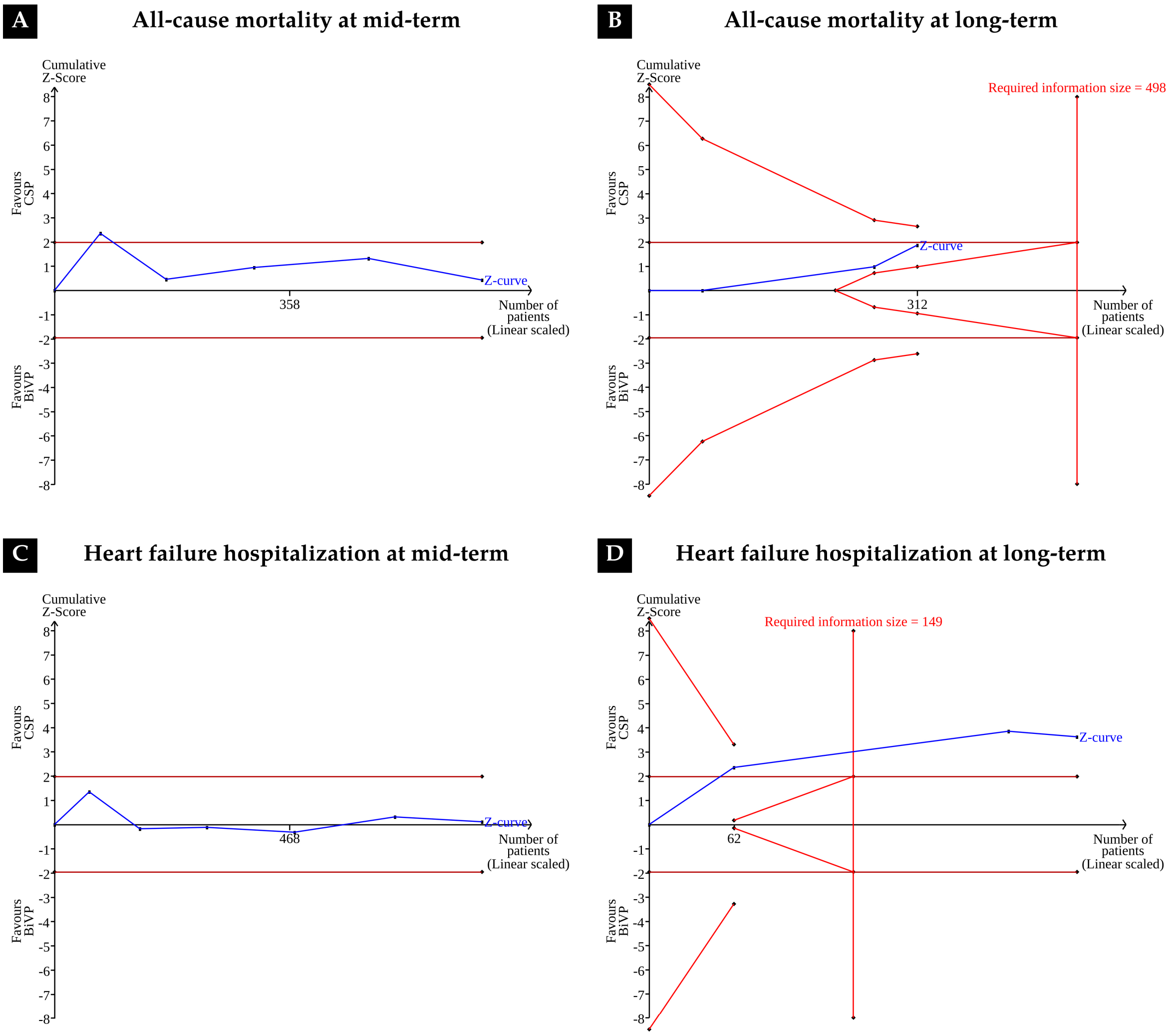


**Supplemental Figure S2.** Trial sequential analysis of effects of CSP versus BiVP on (A) all-cause mortality (ACM) at mid-term (RIS not estimable), (B) ACM at long-term (RIS 498, not attained), (C) heart failure hospitalization (HFH) at mid-term (RIS not estimable), and (D) HFH at long-term (RIS 149, attained; Z-curve crossed the monitoring boundary favoring CSP). BiVP = biventricular pacing; CSP = conduction system pacing; RIS = required information size.

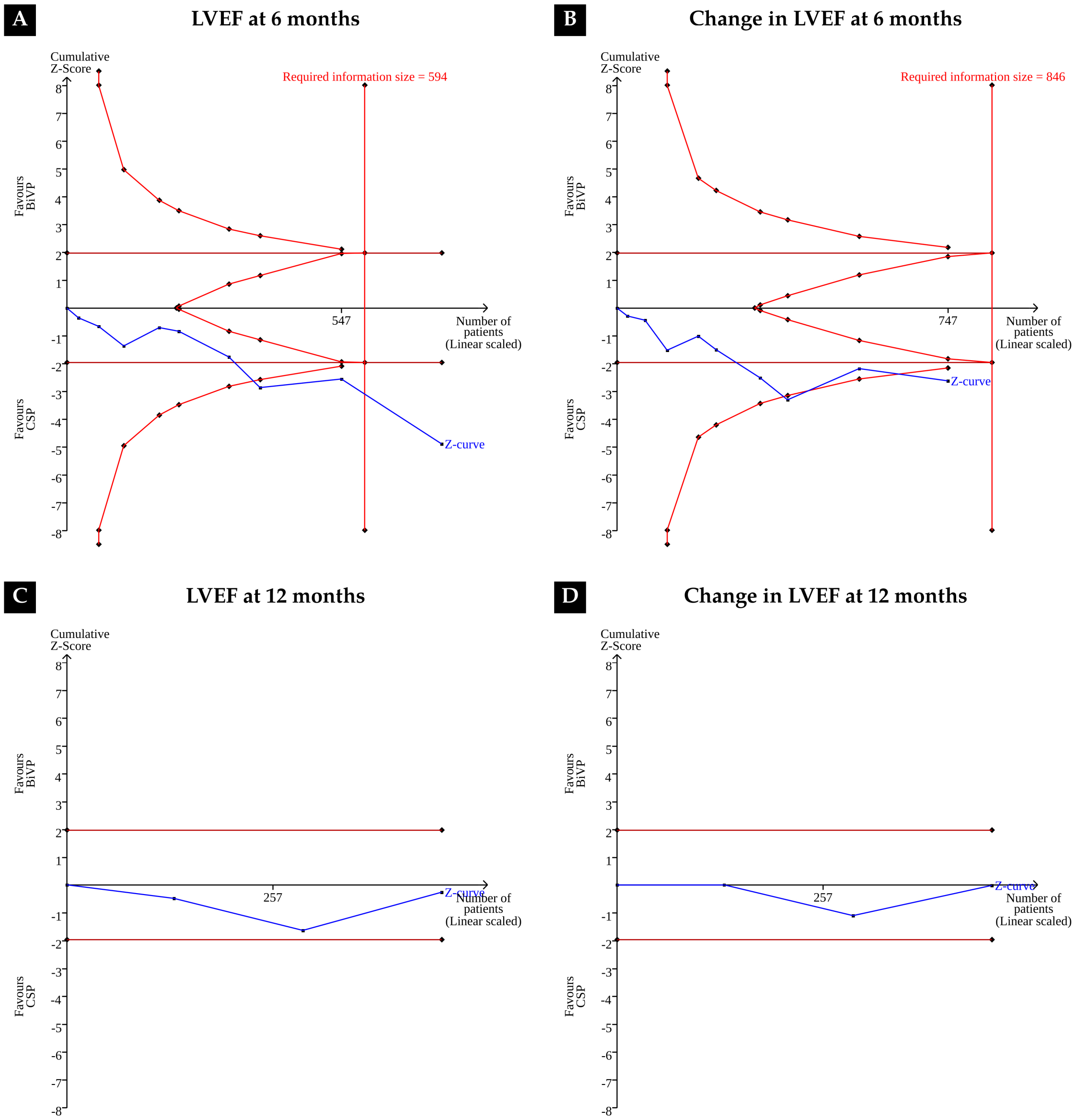


**Supplemental Figure S3.** Trial sequential analysis of effects of CSP versus BiVP on (A) left ventricular ejection fraction (LVEF) at 6 months (RIS 594, attained; Z-curve crossed the monitoring boundary favoring CSP), (B) change in LVEF at 6 months (RIS 846, not attained; Z-curve crossed the monitoring boundary favoring CSP), (C) LVEF at 12 months (RIS not estimable), and (D) change in LVEF at 12 months (RIS not estimable). BiVP = biventricular pacing; CSP = conduction system pacing; RIS = required information size.

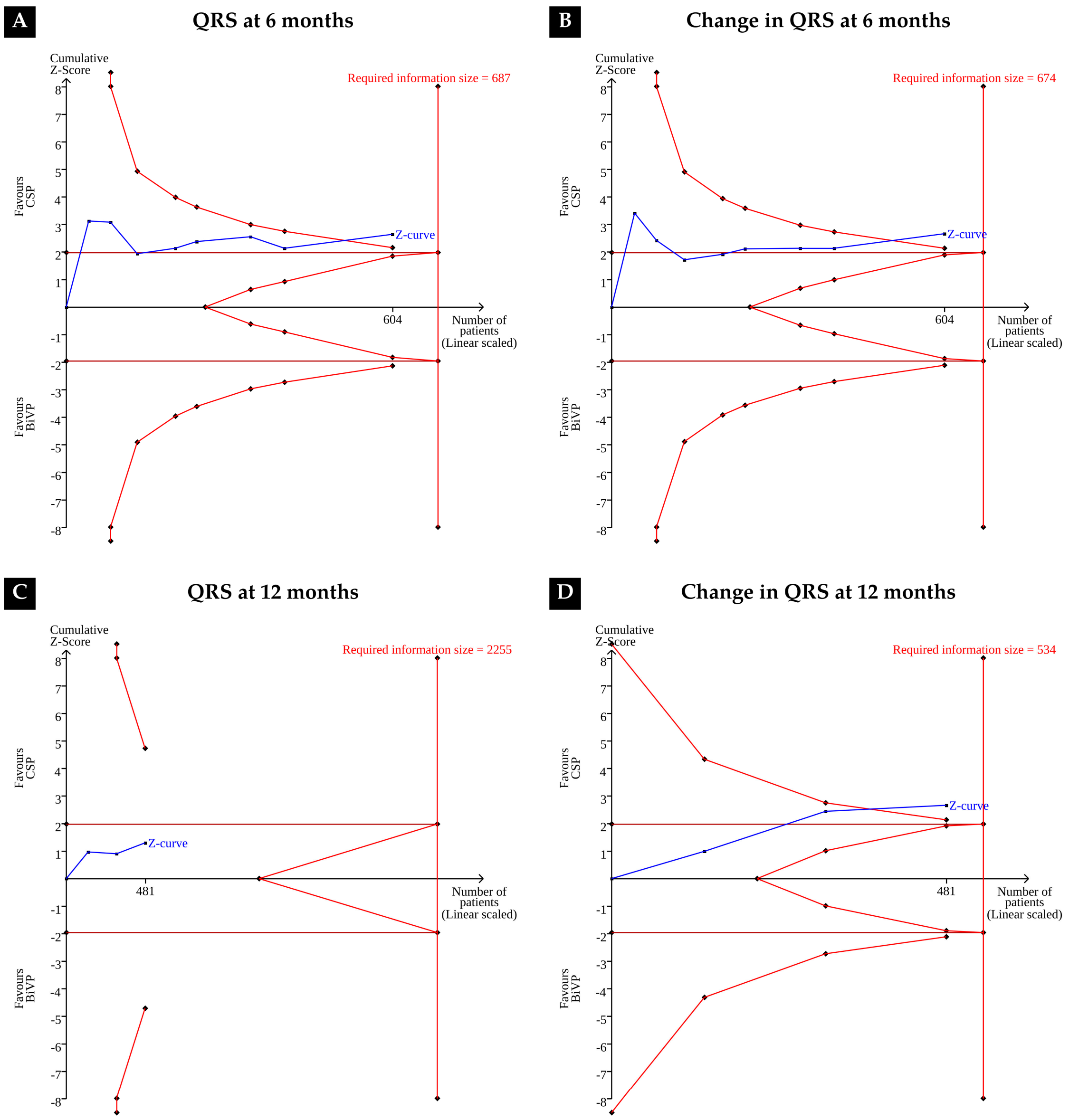


**Supplemental Figure S4.** Trial sequential analysis of effects of CSP versus BiVP on (A) QRS at 6 months (RIS 687, not attained; Z-curve crossed the monitoring boundary favoring CSP), (B) change in QRS at 6 months (RIS 674, not attained; Z-curve crossed the monitoring boundary favoring CSP), (C) QRS at 12 months (RIS 2255, not attained), and (D) change in QRS at 12 months (RIS 534, not attained; Z-curve crossed the monitoring boundary favoring CSP). BiVP = biventricular pacing; CSP = conduction system pacing; RIS = required information size.

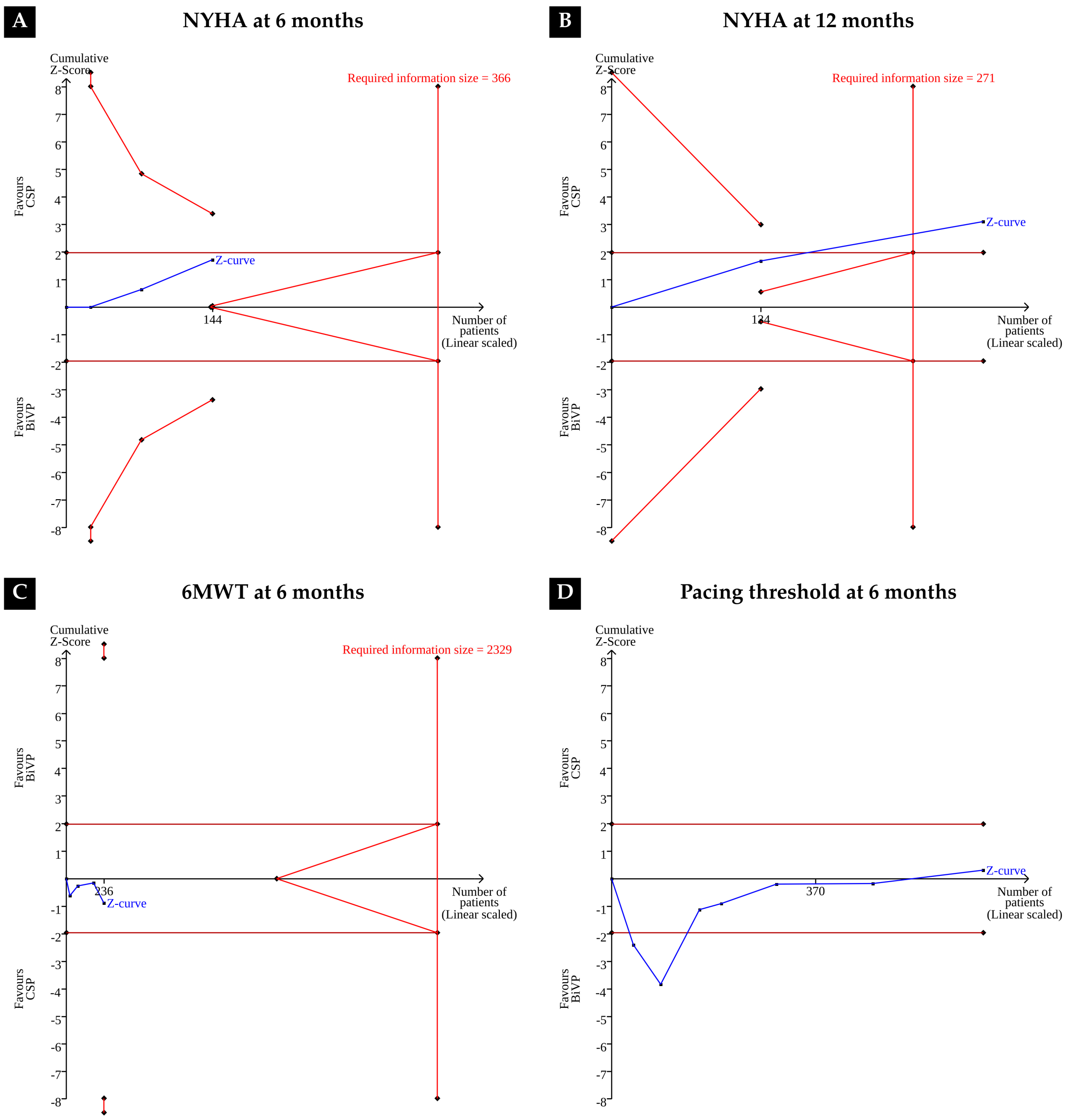


**Supplemental Figure S5.** Trial sequential analysis of effects of CSP versus BiVP on (A) New York Heart Association (NYHA) class at 6 months (RIS 366, not attained), (B) NYHA class at 12 months (RIS 271, attained; Z-curve crossed the monitoring boundary favoring CSP), (C) 6-minute walk test (6MWT) distance at 6 months (RIS 2329, not attained), and (D) pacing threshold at 6 months (RIS not estimable). BiVP = biventricular pacing; CSP = conduction system pacing; RIS = required information size.

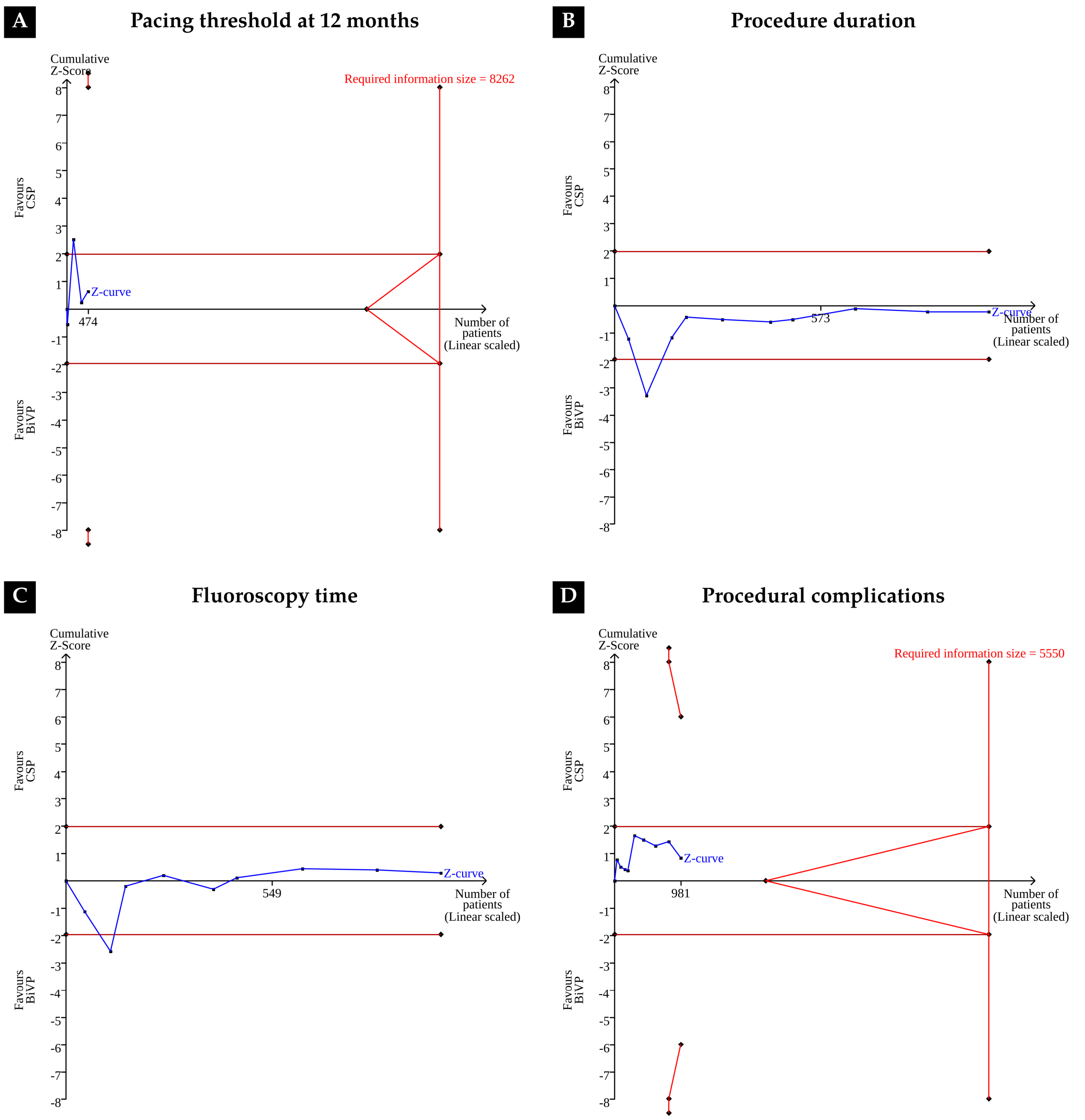


**Supplemental Figure S6.** Trial sequential analysis of effects of CSP versus BiVP on (A) pacing threshold at 12 months (RIS 8262, not attained), (B) procedure duration (RIS not estimable), (C) fluoroscopy time (RIS not estimable), and (D) procedural complications (RIS 5550, not attained). BiVP = biventricular pacing; CSP = conduction system pacing; RIS = required information size.

**Supplemental Figure S7:** PRISMA 2020 checklist.

## PRISMA 2020 Checklist

| Section and Topic | Item # | Checklist item | Location where item is reported |
|---|---|---|---|
| **TITLE** | | | |
| Title | 1 | Identify the report as a systematic review. | Title – identifies the report as a systematic review and meta-analysis ("...A Systematic Review and Meta-Analysis of Randomized Controlled Trials with Trial Sequential Analysis"). |
| **ABSTRACT** | | | |
| Abstract | 2 | See the PRISMA 2020 for Abstracts checklist. | Abstract – structured abstract (Background/Objectives, Methods, Results, Conclusions) reporting eligibility criteria, information sources, synthesis methods, included studies/participants, main results, and limitations. |
| **INTRODUCTION** | | | |
| Rationale | 3 | Describe the rationale for the review in the context of existing knowledge. | Section 1 "Introduction", paragraphs 1–3 – rationale for an updated review given BiVP limitations, the emergence of CSP, and gaps in the prior meta-analysis [12]. |
| Objectives | 4 | Provide an explicit statement of the objective(s) or question(s) the review addresses. | Section 1 "Introduction", final paragraph – explicit statement of objectives and prespecified enhancements (stratification by follow-up duration, subgroup analysis by CSP modality, Trial Sequential Analysis). |
| **METHODS** | | | |
| Eligibility criteria | 5 | Specify the inclusion and exclusion criteria for the review and how studies were grouped for the syntheses. | Section 2.1 "Protocol, Registration, and Study |

# PRISMA 2020 Checklist

| Section and Topic | Item # | Checklist item | Location where item is reported |
|---|---|---|---|
| | | | Eligibility Criteria", paragraph 2 – inclusion and exclusion criteria; grouping for syntheses by follow-up duration and CSP modality described in Sections 2.4 and 2.6. |
| Information sources | 6 | Specify all databases, registers, websites, organisations, reference lists and other sources searched or consulted to identify studies. Specify the date when each source was last searched or consulted. | Section 2.2 "Search Strategy and Study Identification" – MEDLINE (via PubMed), Embase, and Cochrane CENTRAL searched from inception through 15 April 2026; reference lists of included studies and relevant systematic reviews hand-searched. |
| Search strategy | 7 | Present the full search strategies for all databases, registers and websites, including any filters and limits used. | Section 2.2 – full search strategies for all databases provided in Supplementary Material online, Table S1. |
| Selection process | 8 | Specify the methods used to decide whether a study met the inclusion criteria of the review, including how many reviewers screened each record and each report retrieved, whether they worked independently, and if applicable, details of automation tools used in the process. | Section 2.3 "Study Selection and Data Extraction" – duplicates removed in EndNote 2025; two reviewers (J.M.M., C.D.A.) independently screened titles/abstracts and full texts in Rayyan; disagreements resolved by a third reviewer (C.E.S.). |
| Data collection process | 9 | Specify the methods used to collect data from reports, including how many reviewers collected data from each report, whether they worked independently, any processes for obtaining or confirming data from study investigators, and if applicable, details of automation tools used in the process. | Section 2.3 – two reviewers (J.M.M., C.D.A.) independently extracted data into a standardized form; discrepancies resolved by a third reviewer (C.E.S.). |

| Section and Topic | Item # | Checklist item | Location where item is reported |
|---|---|---|---|
| Data items | 10a | List and define all outcomes for which data were sought. Specify whether all results that were compatible with each outcome domain in each study were sought (e.g. for all measures, time points, analyses), and if not, the methods used to decide which results to collect. | Section 2.4 "Study Endpoints" – primary outcomes (ACM, HFH at mid-term/long-term) and secondary outcomes (LVEF, ΔLVEF, QRS, ΔQRS, LVESV, NYHA, 6MWT, pacing threshold, procedure duration, fluoroscopy time, periprocedural complications) fully defined. |
| | 10b | List and define all other variables for which data were sought (e.g. participant and intervention characteristics, funding sources). Describe any assumptions made about any missing or unclear information. | Section 2.3 and Table 1 – study/patient characteristics (age, sex, LVEF, NYHA, QRS, LBBB%, follow-up, GDMT%) extracted and tabulated. Handling of unavailable data (intention-to-treat data preferred; as-treated data used for Upadhyay et al. [26]; both pacing periods of the crossover trial by Lustgarten et al. [25] analyzed as independent groups) described in Section 4, "Strengths and Limitations". |
| Study risk of bias assessment | 11 | Specify the methods used to assess risk of bias in the included studies, including details of the tool(s) used, how many reviewers assessed each study and whether they worked independently, and if applicable, details of automation tools used in the process. | Section 2.5 "Risk of Bias Assessment" – Cochrane RoB2 tool; two independent reviewers (J.M.M., C.D.A.); third reviewer (C.E.S.) resolved discrepancies. |
| Effect measures | 12 | Specify for each outcome the effect measure(s) (e.g. risk ratio, mean difference) used in the synthesis or presentation of results. | Section 2.6 "Statistical Analysis", paragraph 1 – risk ratio (RR) for dichotomous outcomes; mean difference (MD) for continuous |

| Section and Topic | Item # | Checklist item | Location where item is reported |
|---|---|---|---|
| | | | outcomes; standardized mean difference (SMD) for LVESV. |
| Synthesis methods | 13a | Describe the processes used to decide which studies were eligible for each synthesis (e.g. tabulating the study intervention characteristics and comparing against the planned groups for each synthesis (item #5)). | Section 2.4 and the study characteristics in Table 1. |
| | 13b | Describe any methods required to prepare the data for presentation or synthesis, such as handling of missing summary statistics, or data conversions. | Section 2.6, paragraph 1 – use of SMD for LVESV; Section 4, "Strengths and Limitations" – both pacing periods of the crossover trial analyzed as independent groups. |
| | 13c | Describe any methods used to tabulate or visually display results of individual studies and syntheses. | Section 2.6 (implied), Figures 2–7, and Table 1 – forest plots and summary tables used to display individual-study and pooled results. |
| | 13d | Describe any methods used to synthesize results and provide a rationale for the choice(s). If meta-analysis was performed, describe the model(s), method(s) to identify the presence and extent of statistical heterogeneity, and software package(s) used. | Section 2.6, paragraph 2 – random-effects model (restricted maximum likelihood), $I^2$, $\tau^2$, and Cochran's Q (significance threshold $p < 0.10$) per the Cochrane Handbook; analyses in R version 4.5.2 ("meta" package version 8.2.1); TSA via TSA software version 0.9.5.10 Beta (Copenhagen Trial Unit). |
| | 13e | Describe any methods used to explore possible causes of heterogeneity among study results (e.g. subgroup analysis, meta-regression). | Section 2.6, paragraph 2 – prespecified subgroup analysis by CSP modality (LBBAP vs. HBP) with formal subgroup-interaction testing. |
| | 13f | Describe any sensitivity analyses conducted to assess robustness of the synthesized results. | Section 2.6, paragraph 2 – leave-one-out sensitivity analysis |

| Section and Topic | Item # | Checklist item | Location where item is reported |
|---|---|---|---|
| | | | performed for each outcome, and an additional sensitivity analysis excluding the four RCTs that permitted both LBBAP and HBP. |
| Reporting bias assessment | 14 | Describe any methods used to assess risk of bias due to missing results in a synthesis (arising from reporting biases). | Section 2.5 "Risk of Bias Assessment" – addressed via Cochrane RoB2 Domain 5 ("bias in selection of the reported result"), which evaluates risk of bias due to missing/selectively reported results within each trial; per-study Domain 5 ratings detailed in Supplementary Material online, Figure S1. A separate synthesis-level test for missing studies (e.g., funnel plot/Egger's test) was not performed, appropriate given <10 studies per outcome per Cochrane Handbook guidance. |
| Certainty assessment | 15 | Describe any methods used to assess certainty (or confidence) in the body of evidence for an outcome. | Page 3, Section 2.5 (lines 129–131) – certainty of evidence for each outcome assessed using the GRADE framework. |
| **RESULTS** | | | |
| Study selection | 16a | Describe the results of the search and selection process, from the number of records identified in the search to the number of studies included in the review, ideally using a flow diagram. | Section 3.1 "Search Results" and Figure 1 – PRISMA flow diagram of identification, screening, and inclusion. |
| | 16b | Cite studies that might appear to meet the inclusion criteria, but which were excluded, and explain why they were excluded. | Section 3.1 and Figure 1 – exclusion reasons reported by category (conference abstract n = |

| Section and Topic | Item # | Checklist item | Location where item is reported |
|---|---|---|---|
| | | | 8; observational study n = 3; wrong population n = 3; overlapping population n = 2; no follow-up data n = 2; wrong intervention n = 2; no comparator group n = 1). Section 1, final paragraph – exclusion of one RCT that enrolled patients with narrow QRS [18] and of mixed-model repeated-measures outcome data from another RCT [19], with rationale. |
| Study characteristics | 17 | Cite each included study and present its characteristics. | Section 3.2 “Study Characteristics” [13–17,19,25–30] and Table 1. |
| Risk of bias in studies | 18 | Present assessments of risk of bias for each included study. | Section 3.7 “Quality of the Included Studies” – summarized in text (2 of 11 RCTs low risk, 9 some concerns, none high risk); full per-domain RoB2 ratings for each study presented in Supplementary Material online, Figure S1. |
| Results of individual studies | 19 | For all outcomes, present, for each study: (a) summary statistics for each group (where appropriate) and (b) an effect estimate and its precision (e.g. confidence/credible interval), ideally using structured tables or plots. | Figures 2–7 – per-study events/means, SDs, totals, and effect estimates with 95% CI displayed in forest plots. |
| Results of syntheses | 20a | For each synthesis, briefly summarise the characteristics and risk of bias among contributing studies. | Section 3.2 and Table 1 for study characteristics; Section 3.7 for the overall risk-of-bias summary. |
| | 20b | Present results of all statistical syntheses conducted. If meta-analysis was done, present for each the summary estimate and its precision (e.g. confidence/credible interval) and measures of statistical heterogeneity. If comparing groups, describe the direction of the effect. | Sections 3.3–3.4 and Figures 2–7 – pooled RR/MD/SMD with 95% CI and $I^2$ for each synthesis. |

**PRISMA 2020 Checklist**

| Section and Topic | Item # | Checklist item | Location where item is reported |
|---|---|---|---|
| | 20c | Present results of all investigations of possible causes of heterogeneity among study results. | Section 3.5 “Subgroup Analyses” and Figures 2–7 – results of CSP-modality subgroup analyses and interaction p-values. |
| | 20d | Present results of all sensitivity analyses conducted to assess the robustness of the synthesized results. | Section 3.6 “Sensitivity Analyses” – leave-one-out results summarized, with affected outcomes detailed in Supplementary Material online, Table S2; sensitivity analysis excluding the four RCTs that permitted both CSP techniques detailed in Supplementary Material online, Table S3. |
| Reporting biases | 21 | Present assessments of risk of bias due to missing results (arising from reporting biases) for each synthesis assessed. | Section 3.7 “Quality of the Included Studies” – “Nine RCTs showed some concerns in the selection of the reported result” (RoB2 Domain 5), summarizing missing/selective-reporting risk across included trials; full per-study ratings in Supplementary Material online, Figure S1. No funnel plot/Egger’s test was performed at the synthesis level (not indicated, given <10 studies per outcome). |
| Certainty of evidence | 22 | Present assessments of certainty (or confidence) in the body of evidence for each outcome assessed. | Section 3.7 – certainty of evidence per outcome (GRADE) reported in Supplementary Material online, Table S4; also summarized in the Abstract and Section 5 “Conclusions”. |

## PRISMA 2020 Checklist

| Section and Topic | Item # | Checklist item | Location where item is reported |
|---|---|---|---|
| **DISCUSSION** | | | |
| Discussion | 23a | Provide a general interpretation of the results in the context of other evidence. | Section 4 “Discussion” – findings interpreted against the prior meta-analysis (Ferreira Felix et al. [12]) and individual contributing RCTs throughout. |
| | 23b | Discuss any limitations of the evidence included in the review. | Section 4, “Strengths and Limitations” – sample size relative to the TSA-derived required information size; heterogeneity in CSP definition/implantation criteria; moderate-to-substantial statistical heterogeneity; limited evidence for HBP; risk of bias and low or very low certainty for several endpoints. |
| | 23c | Discuss any limitations of the review processes used. | Section 4, “Strengths and Limitations” – variability in follow-up duration across contributing RCTs, handling of crossover and intention-to-treat data, and limited power of subgroup interaction tests discussed. |
| | 23d | Discuss implications of the results for practice, policy, and future research. | Section 4 and Section 5 “Conclusions” – implications for CRT strategy selection (CSP, particularly LBBAP) and the need for larger, adequately powered RCTs with longer follow-up. |
| **OTHER INFORMATION** | | | |
| Registration and protocol | 24a | Provide registration information for the review, including register name and registration number, or state that the review was not registered. | Section 2.1, paragraph 1 – registered in |

## PRISMA 2020 Checklist

| Section and Topic | Item # | Checklist item | Location where item is reported |
|---|---|---|---|
| | | | PROSPERO, registration number CRD420261371613. |
| | 24b | Indicate where the review protocol can be accessed, or state that a protocol was not prepared. | Section 2.1, paragraph 1 – PROSPERO (CRD420261371613). |
| | 24c | Describe and explain any amendments to information provided at registration or in the protocol. | No amendments were made to the registered protocol, as can be verified in the PROSPERO record. |
| Support | 25 | Describe sources of financial or non-financial support for the review, and the role of the funders or sponsors in the review. | "Funding" statement – "This research received no external funding. Publication fees were provided by Sentara Medical Group, Virginia Beach, VA, USA." |
| Competing interests | 26 | Declare any competing interests of review authors. | "Conflicts of Interest" statement – R.B., A.Y., and E.K. disclose industry relationships; other authors report no conflicts related to this manuscript. |
| Availability of data, code and other materials | 27 | Report which of the following are publicly available and where they can be found: template data collection forms; data extracted from included studies; data used for all analyses; analytic code; any other materials used in the review. | "Data Availability Statement" – data used are publicly available, as this is a systematic review; synthesized datasets available from the corresponding author upon reasonable request. |

*From:* Page MJ, McKenzie JE, Bossuyt PM, Boutron I, Hoffmann TC, Mulrow CD, et al. The PRISMA 2020 statement: an updated guideline for reporting systematic reviews. BMJ 2021;372:n71. doi: 10.1136/bmj.n71.
This work is licensed under CC BY 4.0. To view a copy of this license, visit https://creativecommons.org/licenses/by/4.0/
